# Complement activation at the interface between adipocytes and cancer cells drives tumor progression

**DOI:** 10.1172/jci.insight.184935

**Published:** 2025-02-18

**Authors:** Andres Valdivia, Ana Maria Isac, Horacio Cardenas, Guangyuan Zhao, Yaqi Zhang, Hao Huang, Jian-Jun Wei, Mauricio Cuello-Fredes, Sumie Kato, Fernán Gómez-Valenzuela, Francoise Gourronc, Aloysius Klingelhutz, Daniela Matei

**Affiliations:** 1 Department of Obstetrics and Gynecology,; 2 Robert H. Lurie Comprehensive Cancer Center, and; 3 Department of Pathology, Feinberg School of Medicine, Northwestern University, Chicago, Illinois, USA.; 4 Department of Gynecology, School of Medicine, Pontificia Universidad Católica de Chile, Santiago, Chile.; 5 Department of Microbiology and Immunology, College of Medicine, The University of Iowa, Iowa City, Iowa, USA.; 6 Jesse Brown VA Medical Center, Chicago, Illinois, USA.

**Keywords:** Cell biology, Oncology, Adipose tissue, Cancer

## Abstract

The omentum is the primary site of metastasis for ovarian cancer (OC). Interactions between cancer cells and adipocytes drive an invasive and prometastatic phenotype. Here we studied cancer cell–adipocyte crosstalk by using a direct coculture model with immortalized human visceral nondiabetic pre-adipocytes (VNPADs) and OC cells. We demonstrated increased proliferation, invasiveness, and resistance to cisplatin of cocultured compared with monocultured OC cells. RNA sequencing of OC cells from coculture versus monoculture revealed significant transcriptomic changes, identifying over 200 differentially expressed genes common to OVCAR5 and OVCAR8 cell lines. Enriched pathways included PI3K/AKT and complement activation. Lipid transfer into OC cells from adipocytes induced upregulation of complement C3 and C5 proteins. Inhibiting C3 or C5 reversed the invasive phenotype and C3 knockdown reduced tumor progression in vivo. Increased C3 expression was observed in omental implants compared with primary ovarian tumors and C3 secretion was higher in OC ascites from high-BMI versus low-BMI patients. C3 upregulation in OC cells involved activation of the ATF4-mediated integrated stress response (ISR). Overall, adipocyte–cancer cell interactions promoted invasiveness and tumorigenesis via lipid transfer, activating the ISR, and upregulating complement proteins C3 and C5.

## Abstract 2

The main site of metastasis of ovarian cancer is the omentum, where cancer cells interact with adipocytes. In this manuscript, we describe the mechanism by which these interactions alter tumor progression.

## Graphical abstract

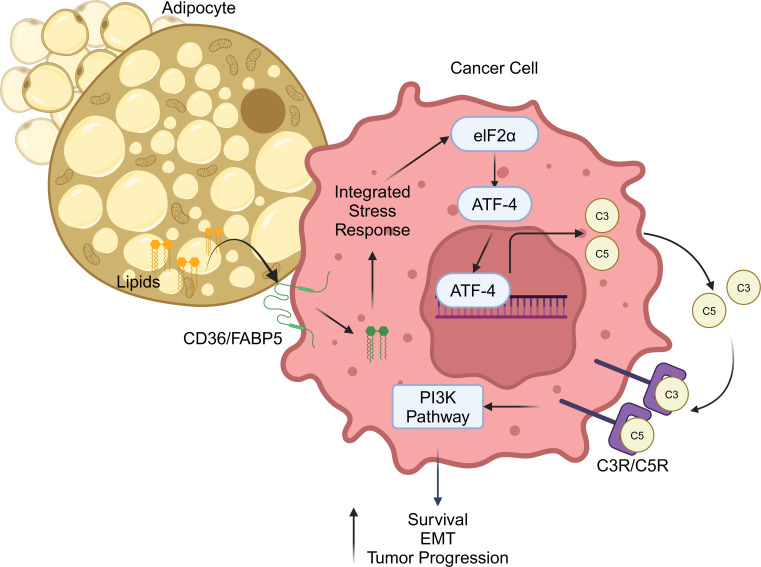

## Introduction

Ovarian cancer (OC) remains a substantial health challenge worldwide, characterized by late-stage diagnosis and tendency to recurrence after initial treatment, leading to high mortality rates (1, 2). The main site of metastasis, the omentum, stands out as a primary metastatic destination due to its unique microenvironment rich in adipocytes (3). It has been speculated that the interactions between OC cells and adipocytes within the omental niche play a crucial role in tumor progression and therapeutic response (4, 5). Lipid transfer from adipocytes to cancer cells mediated by fatty acid binding protein 4 (FABP4) was shown to lead to increased energy generation needed by invasive cells to establish metastasis (6). FABP4 was upregulated in metastases compared with primary tumors, and its forced overexpression in OC cells induced cell migration, invasion, and peritoneal dissemination*,* while its silencing suppressed formation of metastases (5, 7). Further elucidation of the intricate dynamics of cancer cell–adipocyte interactions has been challenging, necessitating innovative model systems that mimic the physiological conditions of the omental microenvironment. In this study, we investigate the crosstalk between OC cells and human immortalized visceral nondiabetic preadipocytes (VNPADs). Our model aims to replicate the direct interactions occurring within the omental metastatic niche and to elucidate the mechanisms driving OC progression and drug resistance.

Previous studies have shown that aside from lipids, adipocytes secrete an array of proinflammatory cytokines, peptide hormones such as leptin, microRNAs, and other factors that act directly on and modify the cancer cells (8–10). Generally, the fatty infiltration of the omentum generates a proinflammatory environment that is conducive to metastatic niche formation and establishment of tumor implants. OC cells expressing the C-X-C motif chemokine receptor 1 (CXCR1) are attracted by IL-6, IL-8, monocyte chemoattractant protein-1, and tissue inhibitor of metalloproteinases-1 secreted by the omentum during tumor dissemination (5). Emerging evidence suggests that the complement cascade, a crucial component of the innate immune system (11), plays a multifaceted role in cancer development and progression (12). Among its various components, complement component 3 (C3) has been studied relative to tumorigenesis and effects on the tumor microenvironment (TME) (13). Complement activation occurs through classical, alternative, or lectin pathways, leading to formation of the C3 convertase complex that ultimately cleaves C3 to active C3a and C3b subunits. In cancer, aberrant activation of the complement cascade was shown to stimulate cancer cell proliferation, invasion, and to promote immune evasion (14). A key study establishing the protumorigenic role of complement comes from the demonstration of enhanced tumor initiation in *Ptx3^–/–^* mice where activation of the complement led to recruitment of tumor-associated macrophages, enhanced inflammation, ultimately promoting oncogenesis (15). Moreover, dysregulation of the complement pathway, particularly increased C3 expression, was associated with poor clinical outcomes in various cancer types (16).

By using a direct coculture model with VNAPDs, we demonstrate increased cell proliferation and resistance to cisplatin in OC cells cocultured with adipocytes. Activation of C3 was identified as a key mediator of these phenotypic changes in OC cells upon coculture with adipocytes. Using preclinical mouse models and human tumor and ascites specimens, we validated that the complement subunit C3 is essential for tumor progression and proliferation of OC cells cocultured with adipocytes. These findings highlight the translational potential of targeting the adipocyte–tumor cell interplay for therapeutic interventions in OC.

## Results

### Coculture of adipocytes and cancer cells.

**Figure 1:**
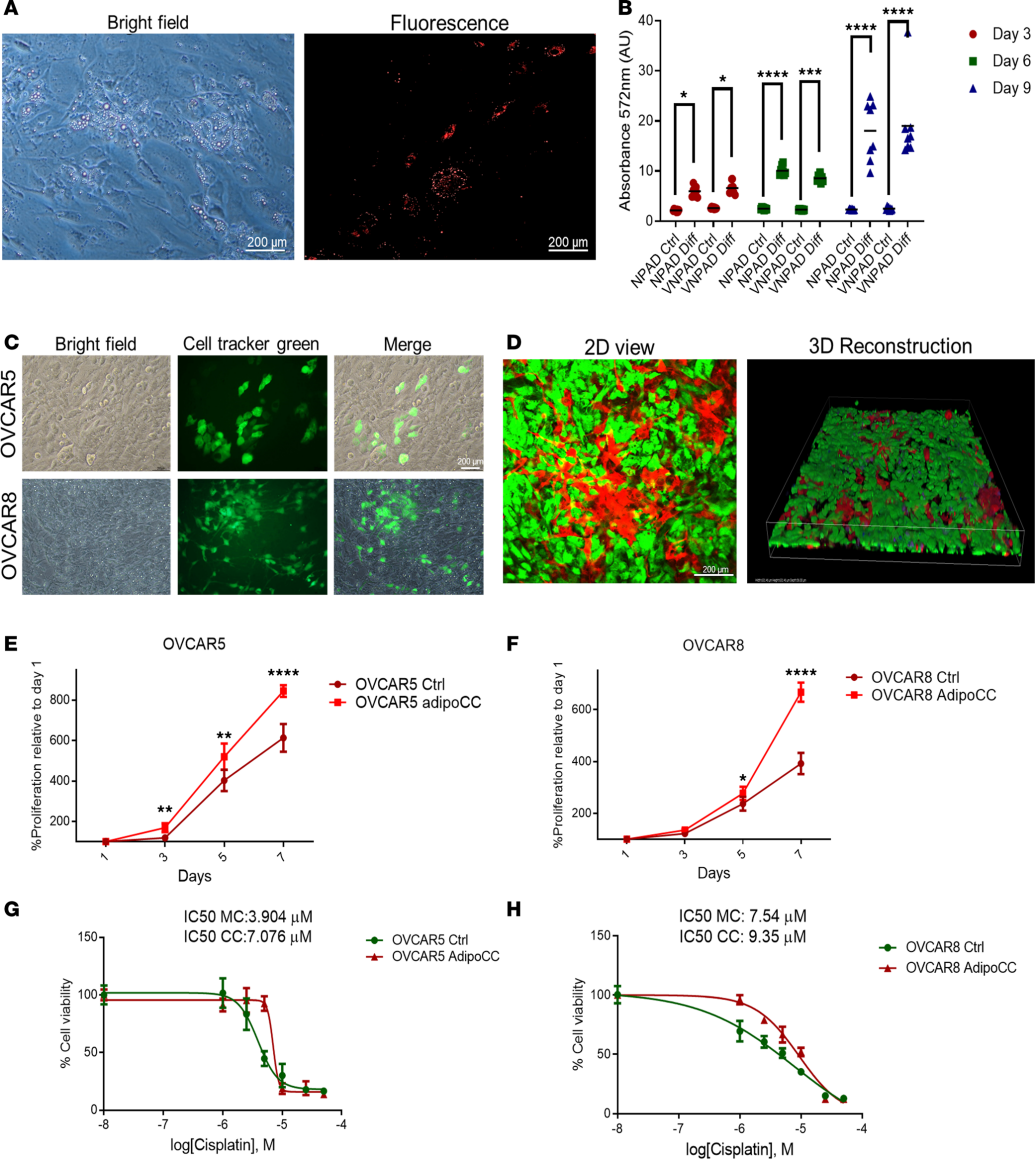
Direct coculture with adipocytes increases proliferation, platinum resistance and in vivo tumor development of OC cells. (**A**) Representative bright-field (left) and fluorescence (right) images of mature VNAPDs stained with AdipoRed on day 9 of differentiation. (**B**) Lipid accumulation measured with AdipoRed on days 3, 6, and 9 after differentiation in NPADs and VNPADs (mean ± SD, *n* = 8). (**C**) Pictures of OVCAR5 or OVCAR8 cells in coculture with VNPADs and stained with CellTracker Green. (**D**) 2D image and 3D reconstruction of an OVCAR8-VNAPD coculture (day 3). OVCAR8 cells and VNPADs were stained with RFP and CellTracker Green, respectively. (**E** and **F**) Proliferation (mean ± SD, *n* = 3) of OVCAR5 (**E**) and OVCAR8 (**F**) OC cells cocultured with adipocytes (AdipoCC) or maintained in monoculture (Ctrl). OC cells were cocultured for 3 days, isolated by FACS, and then cultured to evaluate proliferation on the days indicated using a CCK8 assay. (**G** and **H**) Representative curves of cisplatin effects on viability of OVCAR5 (**G**) and OVCAR8 (**H**) cells following coculture with adipocytes versus the same cell lines maintained in monoculture. *P < 0.05; **P < 0.01; ***P < 0.001; ****P < 0.0001 by 2-way ANOVA with Tukey’s post hoc test (**B**, **E**, and **F**). Scale bars: 200 μm.

To study the interactions between OC cells and adipocytes, we established a long-term coculture model of visceral mature adipocytes, VNPAD-30315 (hereafter referred to as VNPAD) and high-grade serous carcinoma (HGSOC) cells. We first compared the differentiation process of VNPADs with the established NPADs (17, 18). Both cell types were differentiated over a period of 12 days and lipid droplet accumulation was assessed using AdipoRed staining. Accumulation of lipid droplets was observed starting on day 3, reaching maximal quantity on day 12 (Supplemental Figure 1A; supplemental material available online with this article; https://doi.org/10.1172/jci.insight.184935DS1). Lipid droplets were quantified by using AdipoRed staining and spectrophotometry (Figure 1A). VNPADs differentiated and accumulated lipid droplets similarly to the previously described NPADs (17), with progressive increase from day 3 to day 9 (Figure 1B).

To establish a direct coculture model with OC cells, VNAPDs were differentiated for 9 days prior to adding GFP-labeled OVCAR5 or OVCAR8 cells to the culture dish. Figure 1C illustrates that both OVCAR5 and OVCAR8 grow and survive in coculture with adipocytes. Fluorescence microscopy and 3D reconstruction of the coculture shows that OC cells grew on top, on the sides, and underneath the layer of mature CellTracker Green–labeled adipocytes (Figure 1D). After 3 days of coculture with differentiated adipocytes, GFP or CellTracker Green–labeled OC cells were separated by using FACS for characterization. Coculture with visceral adipocytes increased the proliferation of OVCAR5 and OVCAR8 compared with control cells (Figure 1, E and F). Importantly, the increase in proliferation was only observed after coculture with adipocytes, but not after coculture with MET5A mesothelial cells or with undifferentiated VNPADs (Supplemental Figure 1, B and C). Coculture with visceral adipocytes also decreased the sensitivity to platinum in OVCAR5 (from an IC_50_ of 3.904 μM in monoculture to 7.076 μM in coculture, Figure 1G) and OVCAR8 cells (from an IC_50_ of 7.54 μM in monoculture to 9.35 μM in coculture, Figure 1H).

Coculture and treatment with conditioned media from adipocytes promoted OC cell migration (Supplemental Figure 1D). Furthermore, when injected intraperitoneally (i.p.) in nude female mice, adipocyte-cocultured OC cells induced increased tumor weight (Supplemental Figure 1E, *P* = 0.0484) and a trend toward increased tumor volume (Supplemental Figure 1F, *P* = 0.0527) compared with cells maintained in monoculture. There was no statistically significant change in peritoneal tumor numbers derived from adipocyte-cocultured or monocultured OVCAR5 cells in this model (Supplemental Figure 1G).

### RNA sequencing analysis of OC cells cocultured with adipocytes identifies enrichment of complement cascade–related genes.

**Figure 2:**
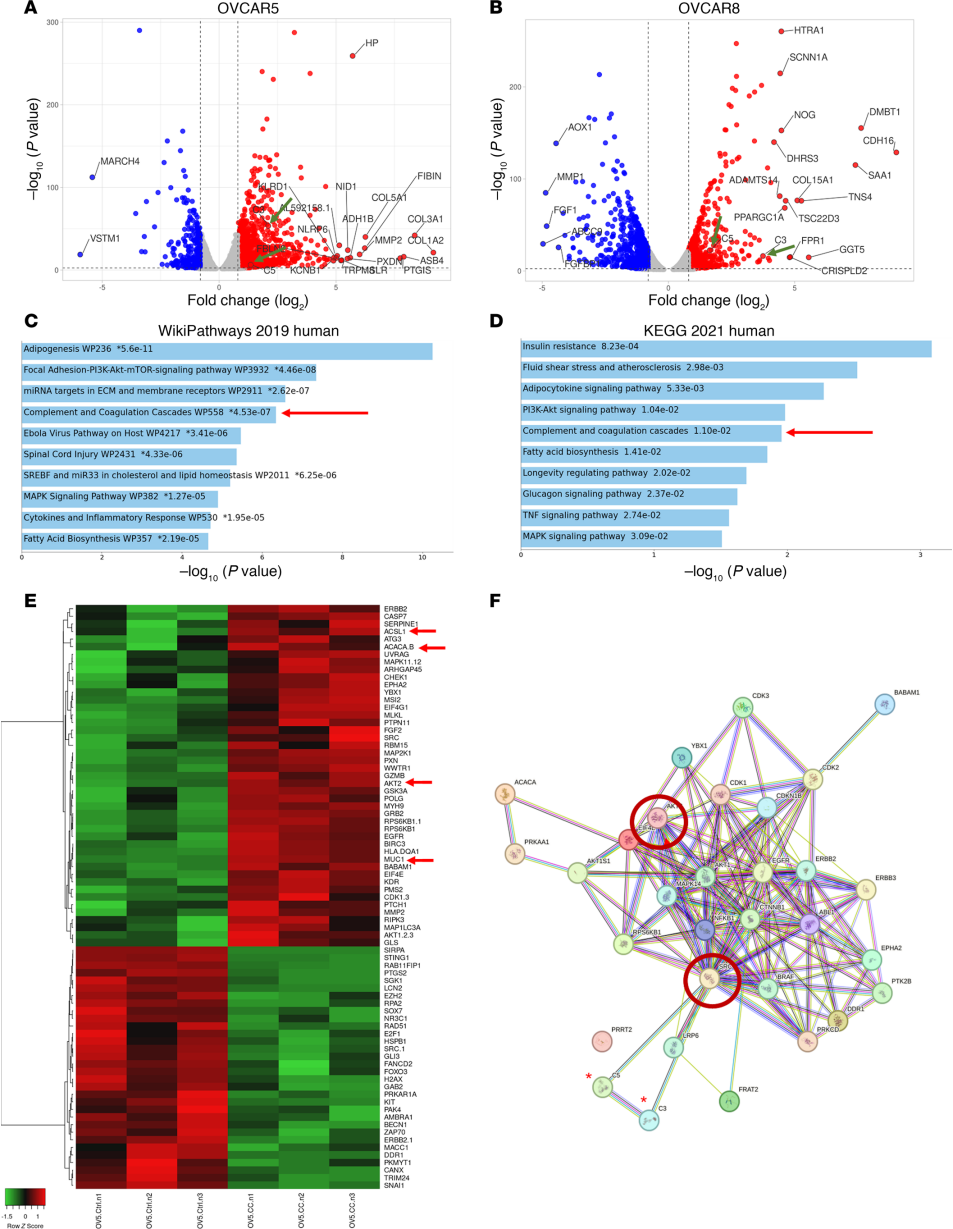
RNA-seq of OC cells cocultured with adipocytes shows enrichment of pathways related to complement activation. (**A** and **B**) Volcano plots show differences in gene expression between OVCAR5 (**A**) and OVCAR8 (**B**) cells cocultured with adipocytes versus cells maintained in monoculture. The green arrows indicate *C3* and *C5* transcripts. (**C** and **D**) Pathway enrichment analysis using the list of DEGs between cocultured versus monocultured OVCAR5 cells (and OVCAR8) performed with Enrichr and the WikiPathways 2019 Human (**C**), or the KEGG 2021 databases. The “Complement and coagulation Cascades” pathway is indicated by red arrows. (**E**) Heatmap of differentially expressed proteins identified by RPPA (*n* = 3 replicates per group). Red arrows indicate proteins also found to be differentially expressed based on RNA-seq analysis. (**F**) String analysis of upregulated proteins. Proteins cluster around SRC and AKT2 (red circles). Complement proteins C3 and C5 (red asterisks) were added to the analysis and predicted to interact with SRC.

To understand the mechanism responsible for the observed change in phenotype, we used an unbiased approach based on RNA sequencing (RNA-seq). OC cells cocultured for 72 hours with mature adipocytes differentiated over 9 days were isolated by FACS in the GFP channel and processed for RNA-seq. A total of 7782 differentially expressed protein-coding genes (DEGs; FDR< 0.05) were identified in OVCAR5 cells cocultured with adipocytes compared with control monocultured cells, of which 4009 transcripts were upregulated and 3773 were downregulated. Similarly, 6246 DEGs, of which 2598 were upregulated and 3102 were downregulated (FDR< 0.05), were identified in OVCAR8 cells cocultured with adipocytes compared with monocultured cells. The top DEGs are included in Supplemental Tables 4 and 5. Overlapping the DEGs between the 2 models, 205 genes were shared between the 2 cell lines (Supplemental Figure 2A). Volcano plots illustrate the DEGs in OVCAR5 (Figure 2A) and OVCAR8 cells (Figure 2B). Transcripts related to the complement cascade (C3 and C5, indicated by the green arrows) were upregulated in both models. Pathway analysis of DEGs in the OVCAR5 model using Metascape, with a cutoff fold change of 1.5 or higher and 0.5-fold or lower, identified enrichment of pathways related to vasculature development and lipid metabolism: “Vasculature development,” “Chemotaxis,” “Lipid localization,” and “Adipogenesis,” among others (Supplemental Figure 2B). The main enriched pathways in the OVCAR8 cell model were related to carbohydrate and lipid metabolism: “Carbohydrate derivate biosynthesis process,” “positive regulation of catabolic process,” “lipid modification,” and “metabolic of lipids,” among others (Supplemental Figure 2C). Pathway analysis using Appyter (19) of the 205 common genes shared between OVCAR5 and OVCAR8 cells identified enriched pathways “Adipogenesis,” “Focal Adhesion-PI3K-AKT-mTOR-Signaling Pathway,” “miRNA Targets in ECM and membrane receptors,” and “Complement and Coagulation Cascades,” among others (WikiPathways 2019 Human, Figure 2C). AKT activation induced by coculture was validated by using Western blotting (Supplemental Figure 2D). KEGG 2021 Human identified as the main enriched pathways “Insulin Resistance,” “Adipokines Signaling Pathways,” “PI3K-AKT Signaling Pathway,” and “Complement and Coagulation Cascades,” among others (Figure 2D). Interestingly, both analyses detected the “Complement and Coagulation Cascades” as being enriched (Figure 2, C and D; red arrows). For OVACAR5, the main regulators controlling the enriched pathways were NFKB1, RELA, SP1, JUN, STAT1, and ATF4 (Supplemental Figure 2E, blue arrows), while for OVCAR8 cells these regulators included HIF1, JUN, EGR1, SP1, and ATF4 (Supplemental Figure 2F, blue arrows). Together, these results indicate that the direct coculture of OC cells with adipocytes generates substantial changes in the OCs cell’s transcriptome.

### RPPA analysis of OC cells cocultured with adipocytes identifies increased phosphorylation of proteins related to the SRC pathway.

To validate the results obtained from the RNA-seq analysis, reverse-phase proteomic analysis (RPPA) was performed. OVCAR5 cells were cocultured with adipocytes for 3 days and isolated by FACS. Of 501 proteins probed, 72 proteins had a change that was statistically significant (*P* < 0.05) — specifically, 45 proteins were upregulated in cocultured cells and 32 proteins were downregulated (Figure 2E and Supplemental Table 6). Of those, several differentially expressed proteins had also been identified as differentially expressed transcripts through RNA-seq (ACSL1, ACACA.B, AKT2 and MUC1, indicated by red arrows in Figure 2E). To better understand the potential interactions between these proteins, we used string analysis (20) for the phosphorylated proteins included in the RPPA. Among the upregulated proteins, we observed previously reported interactions and many proteins clustering around the kinase Src (Figure 2F), EGFR, and the PI3K/AKT pathway (Figure 2F). Because the complement proteins C3 and C5 were not part of the protein set for RPPA, they were added to the pool. String analysis placed C3 and C5 in cluster and close proximity with Src, suggesting that they may be regulated by this kinase. On the other hand, the downregulated proteins clustered around MAPK1, and no interaction was predicted with complement proteins C3 and C5 (Supplemental Figure 2G). Taken together, these data show that coculture with adipocytes increases the expression of proteins from the complement pathway and AKT-regulated pathways.

### Coculture with adipocytes induces expression of the complement cascade proteins C3 and C5.

**Figure 3:**
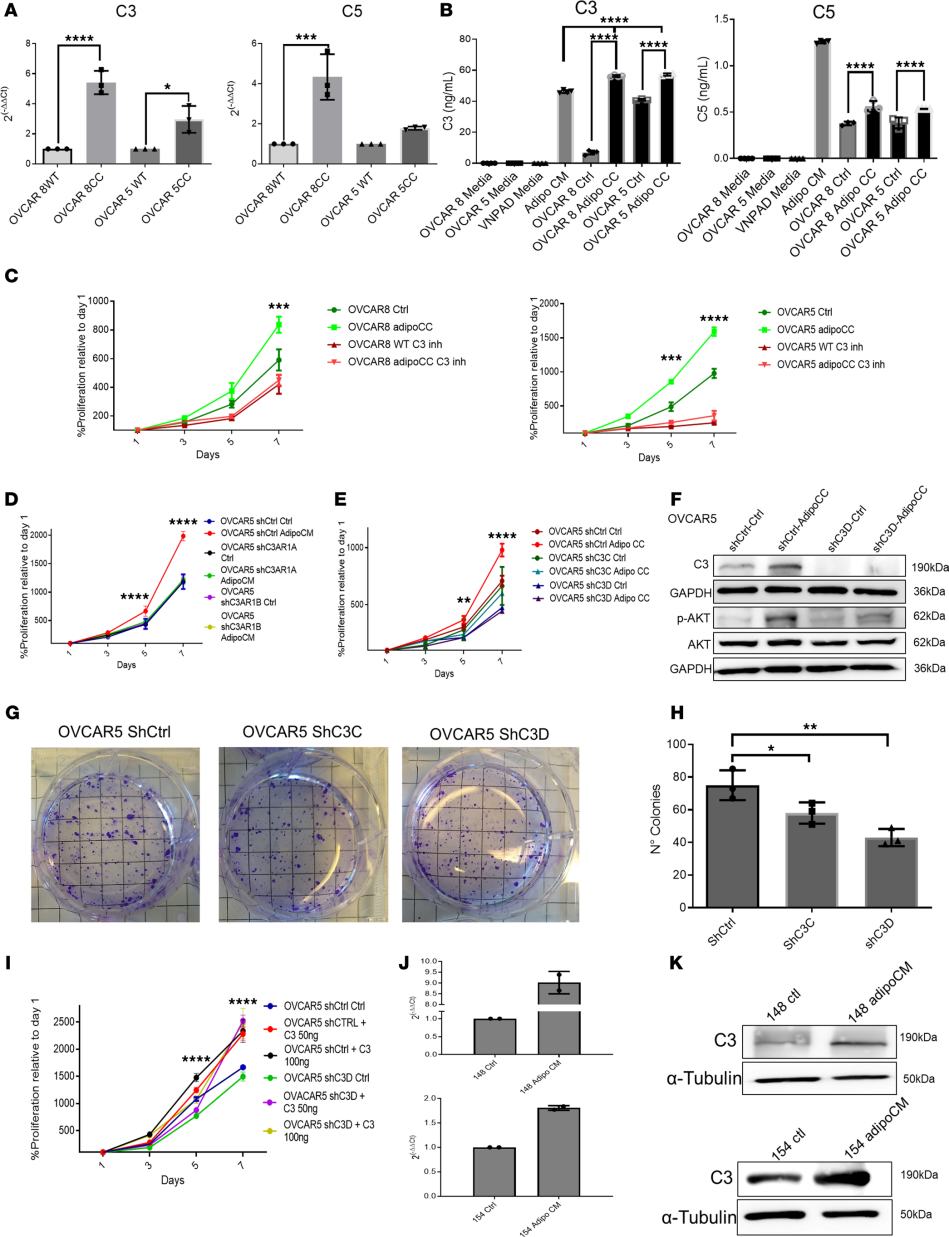
C3 and C5 are overexpressed in OC cells after coculture with adipocytes. (**A**) mRNA expression levels of *C3* and *C5* measured by qRT-PCR in monocultured and cocultured OVCAR8 and OVCAR5 cells (mean ± SD, *n* = 3). (**B**) ELISA-measured protein levels of C3 and C5 secreted in conditioned media of monocultured (Ctrl) and cocultured (Adipo CC) OVCAR8 and OVCAR5 cells (mean ± SD, *n* = 5). Base VNPAD media and adipocyte-conditioned media (Adipo CM) were included as additional controls. (**C**) Proliferation (CCK8 assay) of OVCAR8 (left panel) and OVCAR5 (right panel) cells maintained in monoculture versus cocultured with adipocytes and treated with 240 nM C3R/C5R inhibitor SB290157. (**D**) Proliferation assay of shCtrl and shC3aR1 (clones A and B) OVCAR5 cells cultured in base medium (Ctrl) versus adipocyte-conditioned media (AdipoCM); *n* = 4 replicates. (**E**) Proliferation (CCK8 assay) of shCtrl and shC3 OVCAR5 cells maintained as monoculture (Ctrl) versus cocultured with adipocytes (AdipoCC) (mean ± SD, *n* = 3). (**F**) Western blotting for C3, AKT, and p-Ser^473^ AKT pathway in shCtrl and shC3D OVCAR5 cells grown as monoculture or cocultured with adipocytes. (**G**) Representative picture of colony formation assay of OVCAR5 cells shCtrl and shC3. (**H**) Numbers of colonies (mean ± SD, *n* = 3) determined in a colony formation assay with shCtrl and shC3 OVCAR5 cells. (**I**) Proliferation (CCK8 assay) of shCTRL and shC3 (clone D) OVCAR5 cells cultured either in base medium (Ctrl) or treated with 50 ng or 100 ng recombinant C3. (**J**) qRT-PCR measurements (mean ± SD, *n* = 2) of *C3* mRNA in primary cells derived from human HGSOC tumors (*n* = 2 tumors, nos. 148 and 154) and grown as monoculture or cocultured with adipocytes. (**K**) Western blotting for C3 in the primary OC cells described in panel **I**. *P < 0.05; **P < 0.01; ***P < 0.001; ****P < 0.0001 by 1-way ANOVA with Tukey’s post hoc test (**A**, **B**, and **H**) or 2-way ANOVA with Tukey’s post hoc test (**C**, **D**, **E**, and **I**).

As the complement cascade was identified as being enriched in both OVCAR5 and OVCAR8 cells cocultured with adipocytes, quantitative RT-PCR was used first for validation. The mRNA expression levels of C3 and C5 were increased in OVCAR5 and OVCAR8 cells cocultured with adipocytes (Figure 3A). Coculture with adipocytes also induced increased C3 and C5 secretion into the conditioned media, as measured by ELISA in both cell lines (Figure 3B). To validate the functional importance of the complement cascade activation, we used both a pharmacological inhibitor and shRNA-mediated knockdown. The C3/C5 receptor inhibitor SB290157 reverted the increased proliferative phenotype induced by the coculture with adipocytes in both OVCAR8 (Figure 3C) and OVCAR5 cells (Figure 3C). Similarly, C3a receptor 1 (C3aR1) stable shRNA-mediated knockdown (2 shRNA targeting sequences, Supplemental Figure 3A) prevented the induction in cancer cell proliferation caused by incubation with adipocyte-conditioned media compared with control shRNA–transduced cells (Figure 3D). To further understand the role of C3 in the phenotype of adipocyte-cocultured cancer cells, C3 was knocked down through shRNA-based stable transduction in OVCAR5 cells. C3 expression was statistically significantly reduced in cells transduced with shRNA targeting C3 (2 clones, shC3VC and shC3VD) versus shRNA control (shCtrl; Supplemental Figure 3B). Coculture with adipocytes increased C3 expression in OVCAR5 shCtrl cells, but not in the cells in which C3 had been knocked down (Figure 3F). In parallel, AKT phosphorylation induced by coculture in control cells was prevented in cells in which C3 had been knocked down (Figure 3F).

OVARC5 shC3C- and shC3D-transduced cells cocultured with adipocytes displayed decreased proliferation compared with shCtrl cells (Figure 3E), supporting the notion that C3 is required for the phenotype induced by contact with adipocytes. Likewise, in a colony-forming assay, C3 knockdown led to formation of fewer colonies compared with control-transduced cells (Figure 3, G and H). Addition of recombinant C3 at physiological concentrations rescued the decreased proliferation induced by C3 knockdown (Figure 3I), supporting the concept that the C3 autocrine loop fuels cancer cell proliferation. While recombinant C3 also stimulated the proliferation of adipocytes (Supplemental Figure 3C), it did not affect their differentiation (Supplemental Figure 3D).

Next, we investigated responsiveness to platinum, which had been observed to be decreased in OC cells cocultured with adipocytes. OVCAR5 cells stably transduced with shRNA targeting C3 remained sensitive to cisplatin when cocultured with adipocytes, as opposed to OVCAR5 shCtrl cells which displayed a resistant phenotype (Supplemental Figure 3, E and F), supporting the role of C3 in this process.

To further validate that adipocyte coculture increases C3 expression in OC cells, primary cultures derived from OC-associated ascites were used. Adipocyte coculture increased mRNA (Figure 3J) and protein levels (Figure 3K) of C3 in 2 primary OC cells (*n* = 2). It has been reported that the complement cascade can have either pro- or antitumorigenic roles, depending on the expression of proteins that block the membrane attack complex (MAC) (21). Some of the proteins that prevent the complement MAC formation include C-C motif chemokine ligand (CCL2), complement factor H (CFH), and complement factor H–related 1 (CFH1), among others (15, 22). To determine whether MAC would be formed in our models, we measured the expression of these regulators in cocultured OC cells. CCL2 and CFHR1 were upregulated in cocultured cells, while CFH was downregulated in cocultured OVCAR5 cells (Supplemental Figure 3G), suggesting that MAC formation would be inhibited in this context. Likewise, CCL2 was upregulated, while CFH was downregulated, in OVCAR8 cells (Supplemental Figure 3H). In all, these findings confirm that adipocyte coculture induces the expression of complement cascade proteins C3 and C5, which in turn increase the proliferation of cocultured OC cells.

### C3 knockdown reduces tumor mass and volume in vivo.

**Figure 4:**
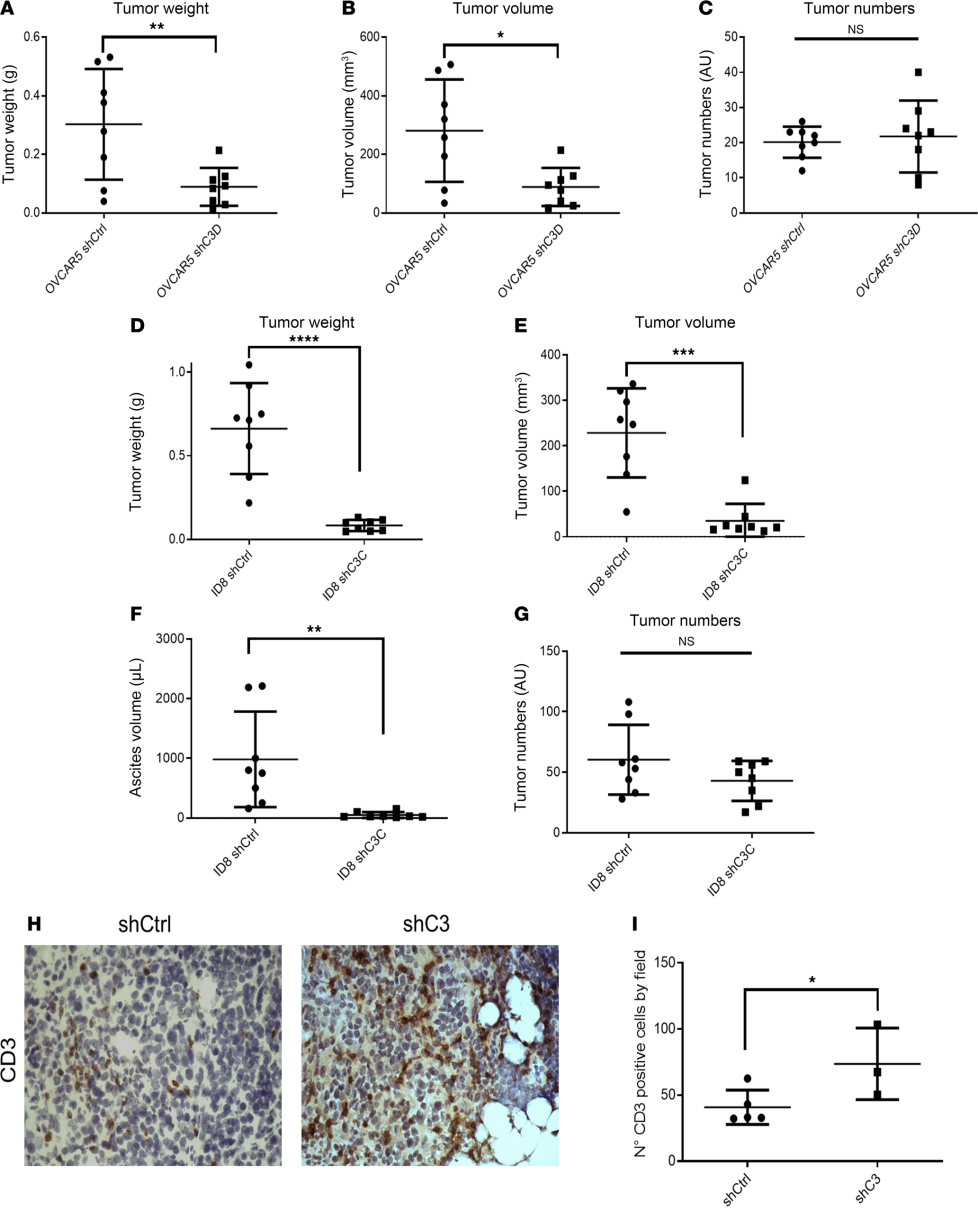
Knockdown of C3 decreases tumor progression of OC cells in vivo. Total tumor weights (**A**), total tumor volumes (**B**), and tumor numbers (**C**) of i.p. xenografts generated from OVCAR5 shCtrl and shC3 cells injected into athymic nude mice (mean ± SD, *n* = 8 per group). Total tumor weights (**D**), total tumor volumes (**E**), ascites volume (**F**), and tumor numbers (**G**) of i.p. xenografts generated from ID8 shCtrl and shC3 cells injected into C57BL/6 immunocompetent mice (mean ± SD, *n* = 8 per group). (**H** and **I**) Representative images of IHC staining (**H**) and quantification of number of CD3^+^ cells (**I**) in the xenografts derived from ID8 cells stably transduced with control shRNA or shRNA targeting C3. Original magnification, ×40. *P < 0.05, **P < 0.01, ***P < 0.001, ****P < 0.0001 by unpaired, 2-tailed Student’s *t* test.

To further study the potential role of C3 in tumor progression, we used i.p. xenografts. After i.p. OC cell inoculation and growth over 4 weeks, smaller tumors were formed in mice injected with OVCAR5 cells in which C3 was knocked down compared with control cells (Supplemental Figure 4, A and B). Tumor weights and volumes generated from OVCAR5 cells in which C3 was knocked down were statistically significantly decreased when compared with control cells (Figure 4A, *P* = 0.009; Figure 4B, *P* = 0.0112, respectively). There was no difference in the number of tumor implants between mice injected with OVCAR5 cells in which C3 was knocked down versus control cells (Figure 4C). Mouse body weights slightly declined after day 20 for mice inoculated with shCtrl cells (Supplemental Figure 4C) and after day 30 in the shC3 group (Supplemental Figure 4D).

Similarly, C3 was knocked down in ID8 cells, a spontaneously tumorigenic mouse ovarian surface epithelial cell line. shRNA-mediated knockdown of C3 in ID8 cells significantly reduced C3 expression in 2 transduced clones (Supplemental Figure 4E). Coculture with adipocytes induced proliferation of shCtrl ID8 cells, but not of ShC3A and shC3C cells, reproducing the phenotype observed with human OC cell lines (Supplemental Figure 4F). Control- and C3 shRNA–transduced ID8 cells were injected i.p. into C57BL/6 immunocompetent mice. Like the OVCAR5 model, tumor weights and volumes generated from ID8 C3-knockdown cells were significantly decreased when compared with controls (Figure 4D, *P* < 0.0001; Figure 4E, *P* < 0.0001, respectively). Mice injected with shCtrl cells harbored significantly more ascites than those injected with shC3C cells (Figure 4F, *P* < 0.001). There was no statistically significant difference in tumor numbers between mice injected with ID8 cells in which C3 was knocked down versus control cells (Figure 4G). Mouse body weights were monitored throughout the experiment and are shown in Supplemental Figure 4, G and H. Given the profound effects of C3 knockdown in the ID8 immunocompetent model, we examined whether antitumor immunity might play a role. Staining for CD3 demonstrated increased infiltration of CD3^+^ cells in C3-knockdown compared with control xenografts (Figure 4, H and I). Collectively, these findings support the notion that C3 is essential for tumor growth and progression.

### C3 expression is induced by lipid uptake in OC cells.

**Figure 5:**
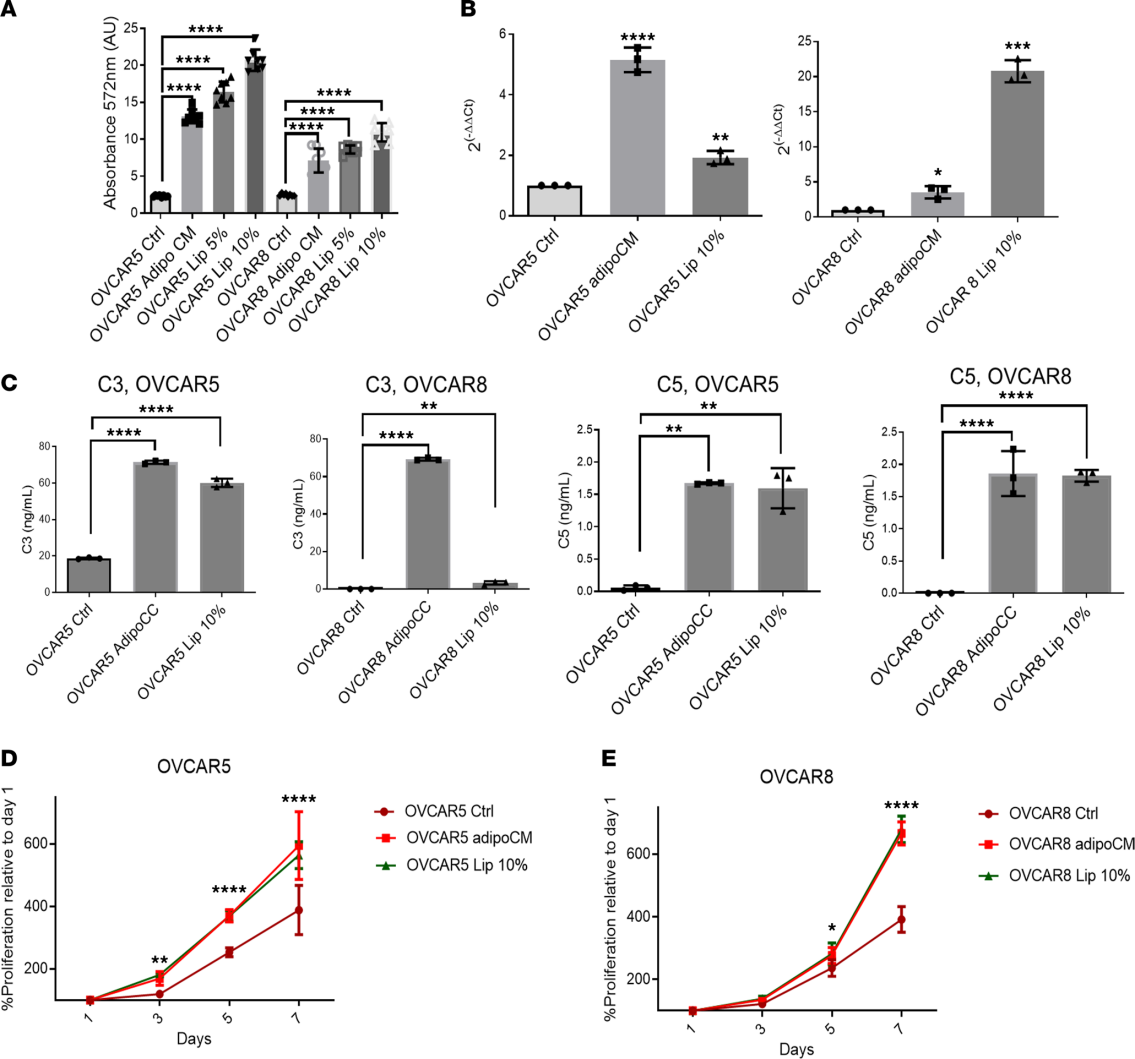
Lipids induce C3 and C5 overexpression and secretion in OC cells. (**A**) Lipid uptake measured via AdipoRed staining (*n* = 6) in OVCAR5 and OVCAR8 cells treated for 3 days with different concentration of lipid mixture or after treatment with adipocyte-conditioned media (Adipo CM). (**B** and **C**) qRT-PCR of *C3* mRNA (*n* = 3) in OVCAR8 and OVCAR5 cells grown for 3 days in media containing 0% or 10% lipid mixture or treatment with adipocyte-conditioned media (Adipo CM). (**C**) C3 (left panels) and C5 (right panels) protein concentrations measured by ELISA (*n* = 5) in media of OVCAR8 and OVCAR5 cells cultured as described in **B**. (**D** and **E**) Proliferation of OVCAR5 (**D**) and OVCAR8 (**E**) cells cultured for 7 days in adipocyte-conditioned media (CM) or media containing 0% or 10% lipid mixture. Values in all panels are mean ± SD. *P < 0.05; **P < 0.01; ***P < 0.001; ****P < 0.0001 by 2-way ANOVA with Tukey’s post hoc test (**A**, **D**, and **E**) or 1-way ANOVA with Tukey’s post hoc test (**B** and **C**).

Adipocytes are the main reservoir of fatty acids in the body and previous studies have shown that transfer of fatty acids and lipids occurs between OC cells and adipocytes (23). To demonstrate that lipid transfer was responsible for the observed C3 induction in cocultured OC cells, the effects of adipocyte-conditioned media (mixed 1:1 with regular media) were compared to a chemically defined lipid concentrate at different concentrations (5%, 10%, or 15% added to regular media). Treatment with both conditioned media from adipocytes or with the lipid mixture induced uptake and accumulation of lipids in OVCAR5 and OVCAR8 cells (Figure 5A). Treatment with both conditioned media or with the lipid mixture induced increased mRNA levels of C3 in OVCAR5 and OVCAR8 cells (Figure 5B) and increased secretion of C3 and C5 measured by ELISA in the conditioned media (Figure 5C). These results confirm that lipid uptake in OC cells induces upregulation of complement elements C3 and C5. Lastly, both conditioned media from adipocytes and the lipid mixture at 10% concentration induced OVCAR5 (Figure 5D) and OVCAR8 (Figure 5E) cell proliferation to levels like those observed in the adipocyte coculture. These findings support the idea that lipid transfer is a key regulator of the phenotype induced by coculture with adipocytes.

### Inhibition of lipid uptake prevents adipocyte coculture–induced cell proliferation and C3 upregulation.

**Figure 6:**
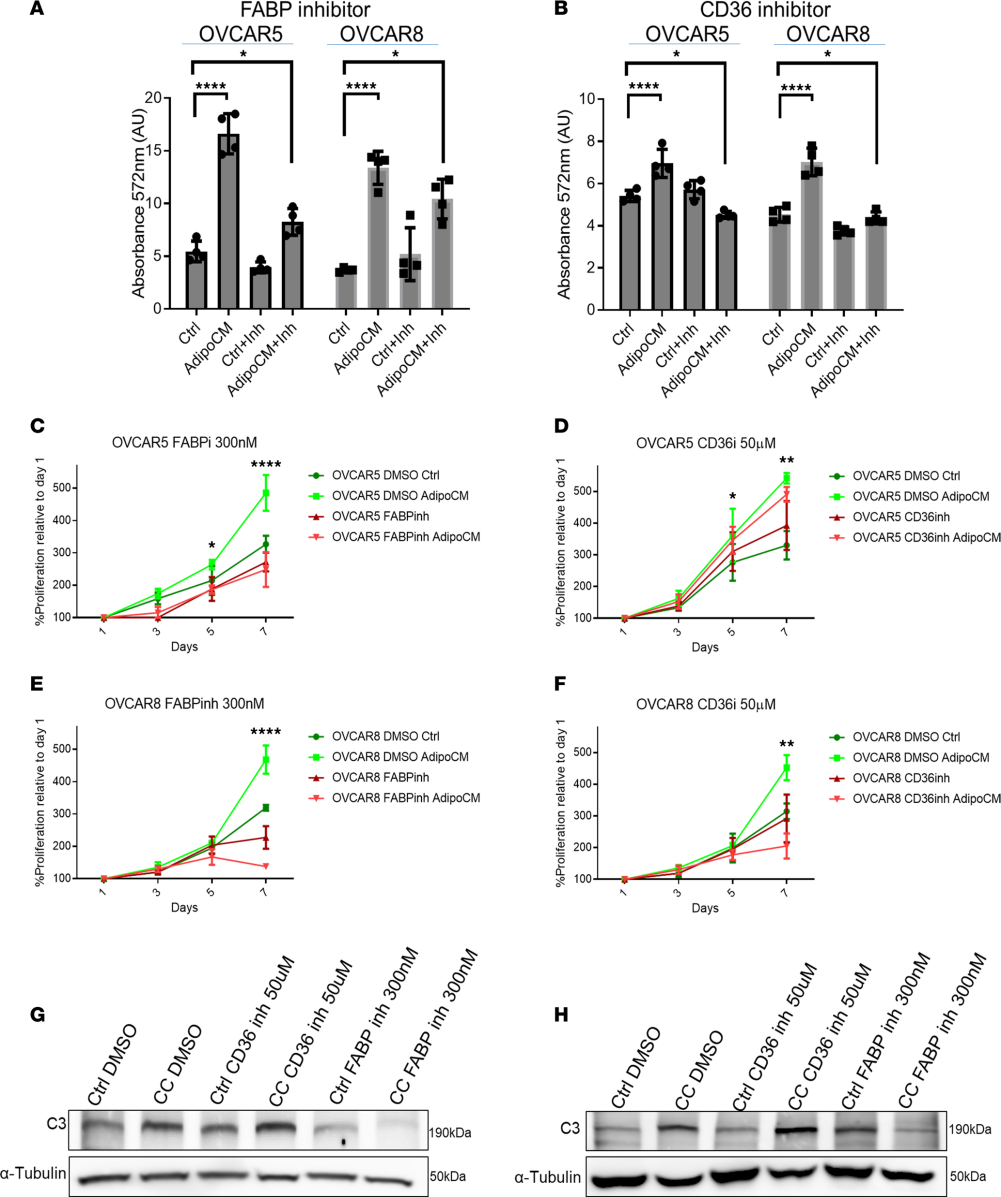
Lipid uptake induces C3 and C5 secretion in OC cells. (**A** and **B**) Lipid uptake determined with AdipoRed in OVCAR8 and OVCAR5 cells cultured in regular or adipocyte-conditioned media (CM) alone or in combination with 20 nM FABP inhibitor (**A**) or 40 μM CD36 inhibitor (**B**) (*n* = 4). (**C**–**F**) Effects of an FABP inhibitor (**C** and **E**) and CD36 inhibitor (**D** and **F**) in OVCAR5 and OVCAR8 cells maintained as monoculture or cocultured with adipocytes (*n* = 3). (**G** and **H**) Western blot analysis of C3 protein levels in OVCAR5 (**G**) and OVCAR8 (**H**) cells maintained as monoculture or following coculture with adipocytes (CC) and treated with FABP or CD36 inhibitor. Values in **A**–**F** are mean ± SD. *P < 0.05; **P < 0.01; ****P < 0.0001 by 2-way ANOVA with Tukey’s post hoc test.

We next used chemical inhibitors of lipid transporters in OC cells. Both the FABP inhibitor (FABPinh, 40 nM) BMS309403 and the CD36 fatty acid receptor inhibitor (CD36inh, 20 μM) sulfosuccinimidyl oleate, significantly reduced lipid uptake in OVCAR5 and OVCAR8 cells (Figure 6, A and B). At these concentrations, the inhibitors were tolerated and nontoxic to cancer cells (Supplemental Figure 5, A and B). Importantly, both inhibitors prevented the increase in cell proliferation induced by adipocyte coculture in OVCAR5 (Figure 6, C and D) and OVCAR8 cells (Figure 6, E and F). Interestingly, only FABP, and not CD36, inhibition prevented adipocyte coculture–induced upregulation of C3 in OVCAR5 (Figure 6G) and OVCAR8 cells (Figure 6H). These findings support the link between lipid uptake, upregulation of C3 secretion, and cell proliferation induced by coculture with adipocytes.

### Adipocyte–cancer cell coculture activates the integrated stress response pathway.

**Figure 7:**
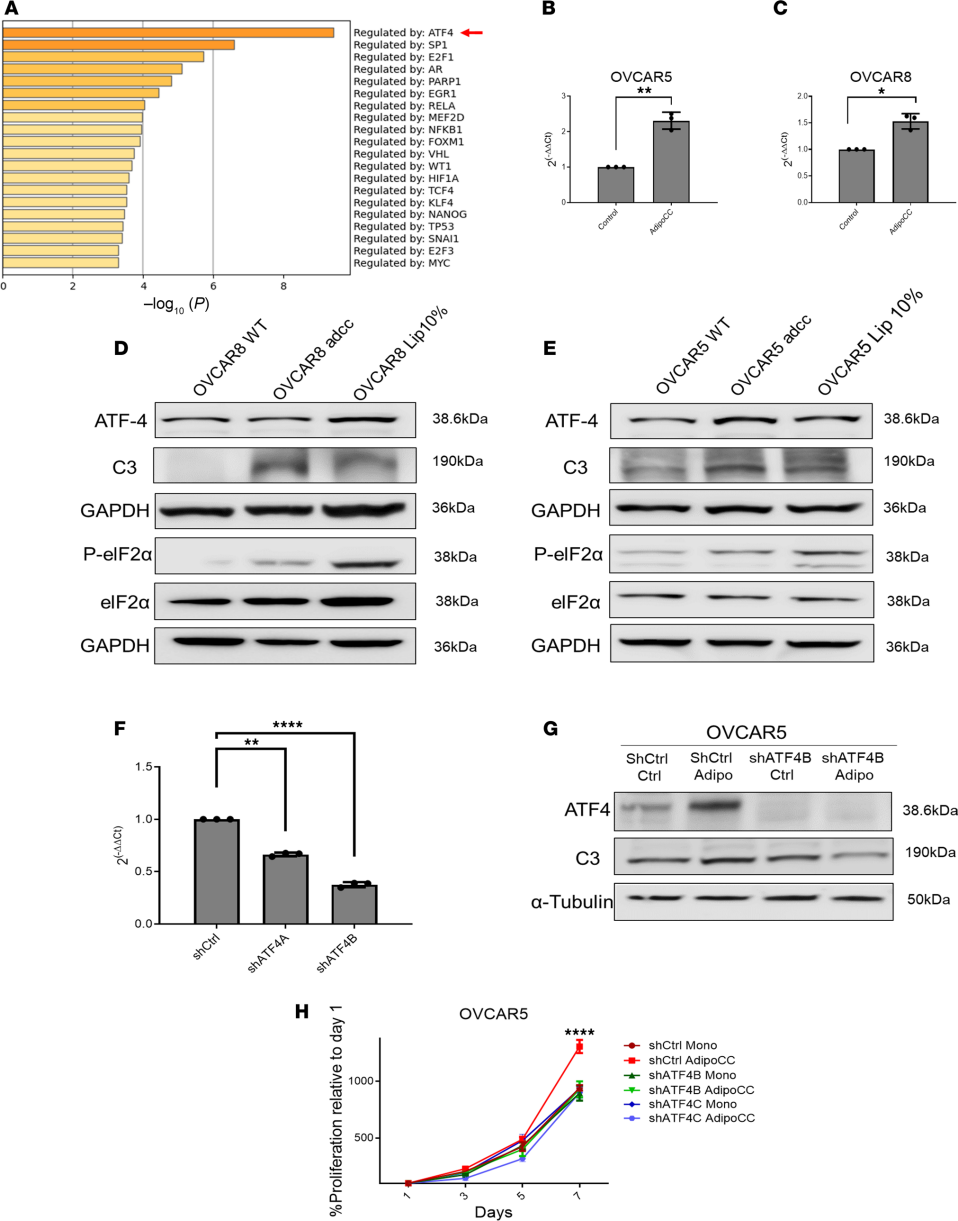
Adipocyte coculture increases activation of the integrated stress pathway response in OC cells. (**A**) Main regulator analysis (Metascape) shows ATF4 (red arrow) as the main regulator of OVCAR5 and OVCAR8 common genes. (**B** and **C**) Effects of coculture of OVCAR5 (**B**) and OVCAR8 (**C**) cells with cocultured adipocytes on mRNA expression levels of ATF4 measured by qRT-PCR (*n* = 3). (**D** and **E**) Western blot of integrated stress pathway response proteins in OVCAR8 (**D**) and OVCAR5 (**E**) cells cultured in regular media supplemented with none or 10% lipid mixture or following coculture with adipocytes. (**F**) shRNA knockdown of ATF4 gene expression verified by qRT-PCR in OVCAR5 cells (*n* = 3). “A” and “B” indicate 2 different shRNAs sequences used. (**G**) Western blot measuring ATF4 and C3 protein levels in shCtrl and shATF4 OVCAR5 cells grown as monoculture or cocultured with adipocytes. (**H**) Proliferation of shCtrl and shATF4 OVCAR5 cells, maintained as monoculture (Mono) or following coculture with adipocytes (AdipoCC) (*n* = 3). ****P < 0.0001 when comparing shCtrl AdipoCC against the other conditions. Values in panels **B**, **C**, **F**, and **H** are presented as mean ± SD. *P < 0.05, ****P < 0.0001 by 1-way ANOVA with Tukey’s post hoc test (**B**, **C**, and **F**) or 2-way ANOVA with Tukey’s post hoc test (**H**).

To understand the mechanisms responsible for induction of C3 secretion and increased proliferation promoted by coculture with adipocytes, the main transcriptional regulators were extracted from the RNA-seq analysis by using Metascape. ATF4 emerged as a key regulator of the common genes in both OVCAR5 and OVCAR8 cells (Figure 7A, red arrow). ATF4 is one the main effectors involved in the cellular response to stress (24), and in the integrated stress response (ISR) pathway. After phosphorylation of elF2α, ATF4 is activated, in turn inducing the expression of prosurvival genes (25). To validate that the ISR was activated in this model, key elements of the pathway were measured. Coculture with adipocytes increased ATF4 mRNA expression levels in OVCAR5 (Figure 7B) and OVCAR8 cells (Figure 7C). Furthermore, adipocyte coculture or addition of the lipid mixture caused increased ATF4 expression and elF2α phosphorylation (Figure 7, D and E). In parallel, C3 was upregulated at the protein level in both OVCAR8 (Figure 7D) and OVCAR5 cells (Figure 7E) cocultured with adipocytes or treated with the lipid mixture. Next, to verify the role of ATF4 in regulating the phenotype induced by adipocyte coculture, the transcription factor was knocked down through stable shRNA transduction (Figure 7F). ATF4 knockdown was confirmed in cells transduced with shCtrl and shATF4B and cocultured with adipocytes for 3 days, with ATF4 being activated by coculture only in control cells. In parallel, Western blotting against C3 showed increased C3 expression in adipocyte-cocultured control cells but not in cells in which ATF4 was knocked down, suggesting a direct link between ATF4 activation and induction of C3 (Figure 7G). Furthermore, coculture with adipocytes increased the proliferation of control cells, but not of cells in which ATF4 had been knocked down (Figure 7H), confirming that activation of the ISR pathway regulated by ATF4 was involved in the increase in proliferation promoted by coculture with adipocytes, partially due to C3 upregulation.

### C3 expression is increased in metastatic implants and in malignant OC–associated ascites.

**Figure 8:**
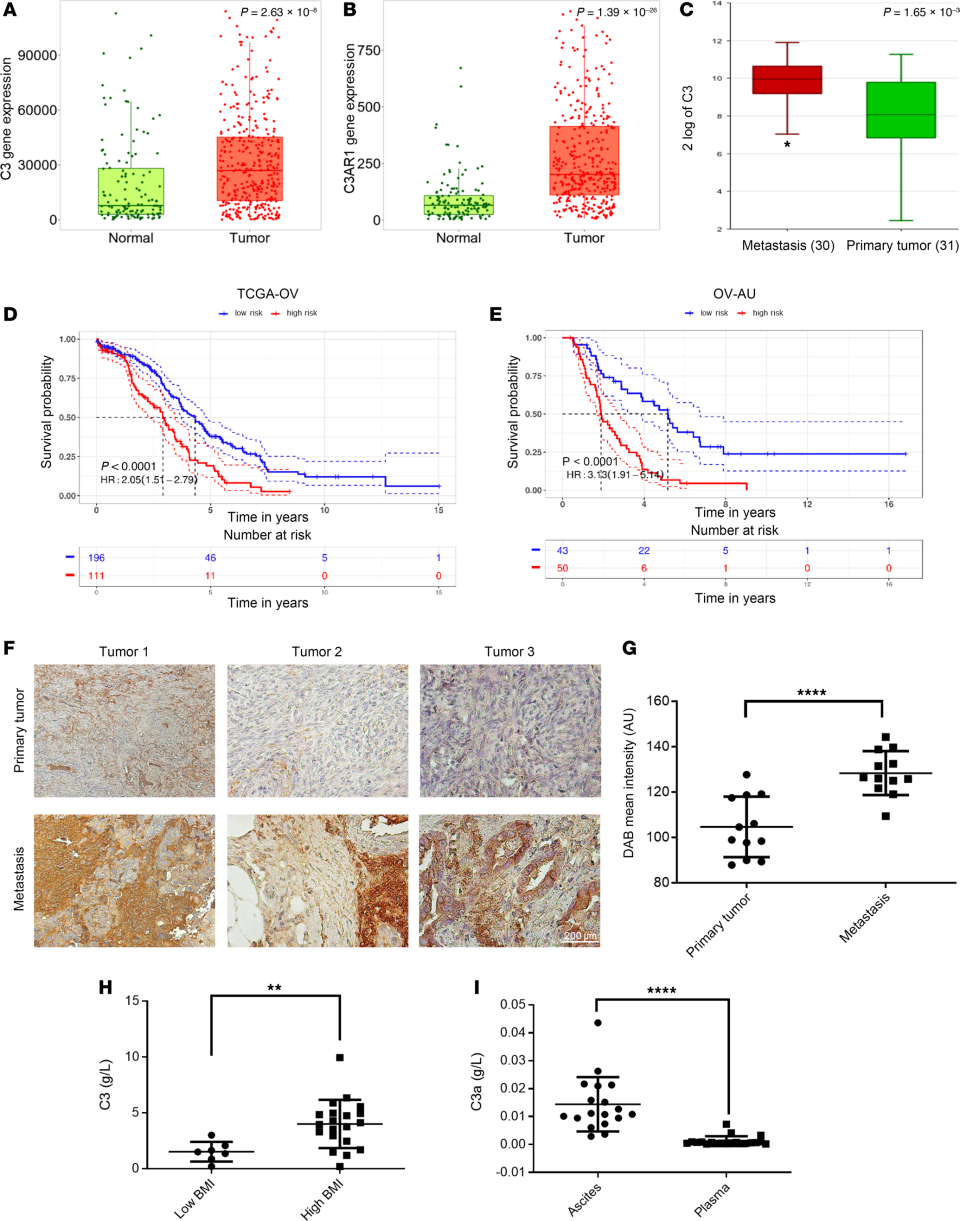
C3 expression in patient tumors increases with contact with omentum and high body mass index. (**A** and **B**) Gene expression analysis by RNA-seq using TNMplot compares *C3* (**A**) and C3A receptor 1 (*C3AR1*) (**B**) in human HGSOC tumors vs. normal ovarian tissue. (**C**) Gene expression analysis by RNA-seq compares *C3* expression between primary tumors against metastatic implants (*n* = 31 paired specimens, GSE204748). The box-and-whisker plots show the median (line within box), IQR (upper and lower box bounds), and 1.5 times the IQR (whiskers). No outliers were found, except for a lower extreme of 7.03 for the metastasis group. (**D** and **E**) Kaplan-Meier overall survival curves determined by multivariate analysis by using combined expression levels of all complement-related genes in TCGA (**D**) and in the Australian ovarian cancer cohorts (**E**) by using the TOPP platform. (**F** and **G**) Representative images of IHC staining (**F**) and mean ± SD DAB mean intensity was calculated using Fiji DAB color deconvolution (**G**) of C3 expression in cancer cells in sections of paired primary and metastatic tumors from women with HGSOC (mean ± SD, *n* = 12 paired specimens). Intense C3 staining is noted in cancer cells adjacent to adipocytes in the omental metastases. Scale bar: 200 μm. (**H**) Measurements of C3 concentrations by ELISA in ascites of OC patients with BMI < 25 (*n* = 7) vs. BMI > 25 (*n* = 20). (**I**) Measurements of C3a concentrations by ELISA in ascites (*n* = 18) vs. plasma (*n* = 18) of paired samples from OC patients. **P < 0.01, ****P < 0.0001 by unpaired, 2-tailed Student’s *t* test (**A**–**C**, **G**, and **H**).

After demonstrating that the coculture of OC cells with adipocytes stimulates cell proliferation via C3 activation in vitro and that C3 is essential for tumor progression in vivo, we sought to determine the relevance of the complement cascade elements’ expression in human tumors. Exploration of The Cancer Genome Atlas (TCGA) HGSOC and GTEx databases by using TNMplot (26) showed that both C3 and its receptor C3R1 were significantly upregulated in HGSOC tumors (*n* = 374) compared with normal ovarian tissue (*n* = 133, *P* = 2.63 × 10^–8^, *P* = 1.39 × 10^–26^, respectively; Figure 8, A and B). C3 was also overexpressed in paired metastatic OC implants compared with primary tumors (*P* = 1.65 × 10^–3^, Figure 8C) based on analysis of the NCBI Gene Expression Omnibus (GEO) publicly available database GSE204748 (27). Multivariate analysis using the TOPP platform (28) and the complement cascade proteins (C1qA, C1qB, C1qC, C1qBP, C1R, C1S, C2, C3, C4A C5, C5R1, CR2, Cr1L CFH, and CCL2, among others) showed that the combined expression of these proteins correlated with worse overall survival, with a hazard ratio of 2.05 for the TCGA cohort (29) (Figure 8D) and 3.13 for the Australian cohort (30) (Figure 8E). These data support the concept that activation of the complement pathway promotes oncogenic pathways relevant to clinical outcomes.

Given that the crosstalk between OC cells and adipocytes is best represented by tumors seeded in the the fat-rich omentum, we evaluated whether C3 is induced in this setting. Comparison of expression of C3 in paired primary versus metastatic omental tumors (*n* = 12 paired specimens) by immunohistochemistry (IHC) revealed significantly higher C3 expression in cancer cells located close to adipocytes compared with cancer cells from primary tumors, as quantified by DAB intensity (Figure 8, F and G; *P* < 0.0001). As C3 is a secreted protein, we sought to determine whether it was detectable in OC-associated malignant ascites and secreted at higher levels in obese patients, who have increased visceral adiposity. ELISA was used to measure C3 levels in OC ascites and plasma from patients with high body mass index (BMI > 25, *n* = 20) versus low BMI (BMI < 25, *n* = 7). As shown in Figure 8H, C3 expression was significantly higher in ascites from high- versus low-BMI patients (*P* < 0.001). Interestingly, the active (cleaved) form of C3 (C3a) was detectable at higher levels in OC-associated ascites compared with plasma from the same patients (Figure 8I). Together, our results demonstrate how the interaction between adipocytes and OC cells causing lipid transfer from adipocytes to cancer cells leads to activation of the ISR pathway, through elF2α phosphorylation and ATF4 activation, increasing the expression of prosurvival genes, such as C3, and activating the AKT pathway.

## Discussion

Our results describe the interplay between OC cells and adipocytes and elucidate how these interactions mediate establishment of metastatic foci in the fat-rich omentum. We identify the ISR pathway and elements of the complement cascade as key regulators of the tumorigenic phenotype induced by the direct contact between OC cells and adipocytes.

The interactions between OC cells and adipocytes have been previously investigated by using indirect coculture models (31) and murine fibroblasts (NIH3T3 cells) as pre-adipocytes (5). These studies found increased tumorigenic properties of cancer cells exposed to conditioned media from adipocytes, such as epithelial-mesenchymal transition, enhanced invasiveness, and acquisition of platinum resistance (5, 32, 33). Targeting adipocyte differentiation has been shown to indirectly alter the phenotype of cancer cells, supporting the role of the tumor milieu in facilitating cancer progression (31). Ladanyi and colleagues demonstrated that OC cells treated with conditioned media from adipocytes upregulate the expression of the fatty acid receptor CD36 and of the transporter protein FABP4, suggesting intracellular mobilization of lipids from adipocytes and transfer of lipids into cancer cells as a key mechanism related to the observed phenotypic changes (6, 23). While indirect coculture models have been instrumental in studying this crosstalk, we aimed to develop a more physiological system that replicates the growth of cancer cells in the omentum. To accomplish this, we used human adipocytes derived from visceral fat and a long-term coculture model in which the 2 cell types were grown in close proximity. This model replicates cohabitation of cells in the same space and was used for coculturing other cell types (34–36). By using this model, we show a significant increase in proliferation and acquisition of platinum resistance in OC cells in the presence of adipocytes and identify global transcriptomic and proteomic changes in cancer cells, which we further characterize. These phenotypical changes underscore the importance of the TME in shaping the cancer cells’ behavior and the response to therapy (22, 33, 37).

Through an unbiased approach, we identified the complement C3 and C5 subunits as being upregulated after the cohabitation of OC cells and adipocytes. C3 and C5 are part of the innate immune system, and their main role is the opsonization of damaged and pathogen-infected cells through formation of the MAC (38, 39). Activation of the complement cascade can follow 3 distinct axes: the classic pathway, the lectin pathway, and the alternative pathway (40). All pathways ultimately lead to hydrolyzation of C3 to C3a and C3b and then the hydrolyzation of C5 to C5a and C5b to generate the MAC (41). The complement cascade is finely tuned by several immunomodulatory molecules such as factor H, factor I, vitronectin, C1 inhibitor, CD35, CD46, CD55, and CD59, which prevent uncontrolled activation. In cancer, the complement proteins can have a dual role, acting both as protumorigenic factors or as elements that can attack and eliminate tumor cells (16, 42). For instance, previous reports documented secretion of complement C3a and C5a proteins and activation of their receptors C3R1 and C5R1 in association with cancer progression (43–46). C3a and C5a secretion from OC cells was shown to activate an autocrine loop converging on Akt and stimulating cancer cell survival (45). The experiments presented here show that C3 knockdown decreases cell proliferation induced by coculture with adipocytes and inhibits tumor growth in vivo in xenograft and in syngeneic models. Similarly, C3aR1 knockdown or pharmacological inhibition blocked cancer cell proliferation induced by incubation with adipocyte-conditioned media. These data support the notion that activation of the complement triggered by the interactions with the adipose environment drives OC progression and propose C3 as a potential tumor target. Complement activation also plays a role in the immune cells in the tumor milieu. It has been shown that CD4^+^ T cells store intracellular fragments of C3, which are secreted and activate the C3Rs on the cell surface (46). This interaction is regulated by complement regulators CD46, CD55, and CD59 (47). Complement activation in the TME stimulates proinflammatory cytokine production; however, complement does not typically recognize tumor antigens as targets for attack (42). We observed increased CD3^+^ T cell infiltration in tumors in which C3 was knocked down, supporting an effect of the pathway on modulation of antitumor immunity.

Importantly, the analysis of tumor specimens included here revealed direct correlations between C3 expression, BMI, and disease outcome. We observed increased expression levels of C3 in omental implants compared with primary tumors, suggesting a potential role for C3 in promoting metastasis. Additionally, the combined expression of elements of the complement pathway was associated with overall survival in 2 publicly available transcriptomic databases, supporting the clinical relevance of this pathway to OC. Moreover, we found increased C3 secretion in malignant ascites in patients with high BMI, reinforcing the idea that adipose tissue–derived factors play an important role in shaping the TME and modulating cancer cell behavior (48, 49). As obesity was recognized as a risk factor for cancer (50, 51), our findings provide a potential mechanistic link between visceral fat and aggressive cancer phenotype. These results also suggest that targeting the complement pathway by inhibiting receptors C3R1 and C5R1 could induce potent antitumor effects. Previous studies have suggested that complement receptor inhibitors such as SB290157 inhibit tumor growth in pancreatic (52) and liver cancer (53) models and our findings support further investigation of such receptor antagonists in HGSOC.

The increased expression and secretion of C3 and C5 has been associated with activation of proinflammatory signaling and the ISR pathway, which protects the cells against stress (54–57). Central to this pathway is the activation of the transcription factor ATF4. When cells undergo stress, they activate various survival mechanisms (58), including phosphorylation of elF2α and upregulation of ATF4, which in turn activates prosurvival genes (24, 59). Our findings show that the crosstalk between OC cells and adipocytes triggers an adaptive cellular response mediated by ATF4, which induces C3 expression and promotes cell survival and resistance to cisplatin. The increase in cellular stress may be due to the transfer of lipids from the TME into cancer cells. It had been shown that intake of saturated fatty acids activates the unfolded proteins response and ISR signaling (60–62). Coculture with adipocytes was shown by others to activate oxidative and ER stress (63). These findings expand our understanding of the molecular pathways involved in the crosstalk between OC cells and adipocytes and highlight new potential targets for intervention. Inhibition of phosphoserine aminotransferase 1 (PSAT1), a downstream target of ATF4, was proposed in estrogen receptor–negative breast cancer (64). The coiled-coil domain containing 106 (CCDC106), another ATF4-regulated molecule, has also been suggested as a potential target in OC (65). While our study provides evidence that the secretion of complement proteins and activation of the ISR pathway drives OC progression and platinum resistance, further elucidation of the specific signaling pathways downstream of ATF4 and the ISR, as well as the precise mechanisms by which complement factors influence cancer cell behavior, will be critical for the development of novel therapeutic strategies.

In all, our findings highlight the interplay between OC cells and the adipose microenvironment, providing insight into the molecular mechanisms underlying tumor progression and therapeutic resistance. By identifying key regulators such as ATF4 and complement C3, activated at the interface with the fatty milieu, we propose these targets for therapeutic development.

## Methods

### Sex as a biological variable.

Human specimens were from female patients and animal experiments used female mice, due to ovarian cancer being restricted to women.

### Cell lines and reagents.

OVCAR8 cells were purchased from the ATCC and OVCAR5 cells were obtained from Marcus Peter, Feinberg School of Medicine, Northwestern University. Subcutaneous NPADs and VNPADs, isolate VNPAD-30315, have been previously described (66, 67). Briefly, primary preadipocytes were immortalized by transduction of hTERT and HPV-16 E6/E7 retroviruses, followed by selection. Clones of the cells that retained high differentiation ability, namely, clones B and D for NPAD and VNPAD, respectively, were utilized for this study. Murine surface ovarian epithelial ID8 cells were obtained from Katherine Roby (Department of Cell Biology and Physiology, University of Kansas, Kansas City, Kansas, USA). ID8 stably transduced with shRNA targeting C3 were generated from the original ID8 cells. MET5A cells were obtained from ATCC. All cell lines were cultured at 37°C, 5% CO_2_, and 100% humidity and used for experiments at low passage number (<10). Cell culture media are listed in Supplemental Table 1. Cells were regularly tested in house for mycoplasma contamination using a PCR-based method (ATCC Universal Mycoplasma Detection kit) and by Charles River Animal Diagnostic Services. Cell lines were authenticated by IDEXX BioAnalytics with short tandem repeat (STR) profiling. AdipoRed (catalog PT-7009) was obtained from Lonza. The inhibitor SB290157 (catalog SML1192-5MG) was obtained from Sigma-Aldrich. Chemically Defined Lipid Concentrate (catalog 11905031) was obtained from Thermo Fisher Scientific. The FABP inhibitor BMS309403 (catalog HY-101903) and the CD36 inhibitor sulfosuccinimidyl oleate (catalog 11211-10mg) were obtained from MedChemExpress. Recombinant C3 was from Abcam (catalog ab288822). A list of antibodies, primers, and their sources are included in Supplemental Tables 2 and 3.

### Human specimens.

Fresh deidentified specimens of advanced high-grade serous ovarian tumors and malignant ascites associated with OC were collected from consenting individuals and were immediately processed through enzymatic and mechanical disassociation into a single-cell suspensions, following established procedures (68) to be utilized in various experiments. Paraffin-embedded, deidentified and paired HGSOC specimens, including both primary tumor and omental metastases, were used for IHC. Ascites and plasma samples were collected from patients with advanced-stage HGSOC at the time of diagnosis (needle biopsy guided by CT scan or collection through laparoscopy) or at primary debulking surgery at UC-Christus Health Network, Santiago, Chile. Upon collection, ascites and blood samples were centrifuged at 2500*g* for 15 minutes and aliquots stored at –80°C until analysis.

### Transduction.

Stable gene knockdown was performed by transducing cells with lentiviral particles encoding shRNAs targeting genes of interest. Lentiviral vectors containing nontargeting shRNAs were used for control cells (shCtrl). Lentiviral vectors for shRNA knockdown of C3 (shC3) and ATF4 (shATF4) were purchased from Origene (catalog TL314318V and TL306520V, respectively). Cells were transduced using viral particles at an MOI of 5 over a period of 24 hours. Cells were then selected using 1 μg/mL of puromycin for a period of 28 days. Gene knockdown was verified by GFP fluorescence, RT-PCR, and/or Western blotting.

### Adipocyte differentiation.

NPADs and VNPADs were differentiated into mature adipocytes as described previously (17). Briefly, pre-adipocytes were seeded on a 10-cm dish and cultured in pre-adipocyte growth media (Cell Applications, catalog 811-500) until they reached 80% confluence, and then changed to adipocyte differentiation media (Cell Applications, catalog 811D-250). Adipocyte maturation was assessed by AdipoRed staining of lipid droplets followed by examination with a fluorescence microscope. Mature adipocytes were used for coculture experiments and production of conditioned media. For AdipoRed staining, cells were cultured in 96-well dishes until confluent, washed with PBS, and incubated with 5 μL of AdipoRed mixed in 200 μL of PBS for 10 minutes under culture conditions. Fluorescence was measured with a Spectramax Gemini XS reader set at 485 nm and 572 nm for excitation and emission wavelengths, respectively. Data were analyzed and plotted using GraphPad Prism 6.

### Fluorescence microscopy and image processing.

Images of fluorescent cells or cells stained with the CellTracker Green CMFDA dye (Invitrogen, catalog C7025) were acquired using a Nikon A1 confocal microscope system. The wavelength settings (excitation/emission) used for Alexa Fluor were 488/525 nm for the green channel and 568/561 nm for the red channel. A 3D reconstruction of cocultured cells was performed by applying the *Z*-stack function of the software with a *Z*-step of 2 μm. Image enhancement, including adjustments to brightness and contrast at every pixel, and fluorescence quantification were performed using Fiji software (https://imagej.net/software/fiji/downloads).

### IHC.

IHC was performed on paraffin-embedded human HGSOC specimens using an anti-C3 antibody (catalog PA5-21349) from Invitrogen. IHC for CD3 was performed on frozen xenografts with anti-CD3 (SP7 clone) antibody (Invitrogen, catalog MA5-14524) at the Northwestern University Mouse Histology and Phenotyping Laboratory. Further details can be found in the Supplemental Methods. IHC staining intensity was quantified by DAB deconvolution performed with the Fiji software.

### Adipocyte–cancer cell coculture.

VNPADs and OC cancer cells OVCAR5-GFP and OVCAR8 labeled with CellTracker were used for coculture experiments. Cancer cells (1, 2, or 3 million) were seeded on 10-cm dishes already containing a monolayer of mature adipocytes. Cells were grown in coculture for 3 days and were subsequently detached and sorted by FACS (FACSMelody, BD Bioscience) using the GFP channel for subsequent experiments.

### Cell proliferation assay.

Cells were seeded in 96-well plates at 500 cells per well. Cell proliferation was estimated by using a CCK8 kit (APExBIO, catalog K1018) as described in the manufacturer’s protocol. Absorbance at 450 nm was measured with a microplate reader (BioTek ELX800).

### IC_50_ determinations.

The inhibitory effects of different compounds on cell proliferation were measured in dose-response experiments for calculation of IC_50_ values. Cell proliferation was estimated by determining cell viability using a CCK8 kit as described previously (69). IC_50_ values were determined by logarithm-normalized sigmoidal dose curves fitting using GraphPad Prism 6.

### RNA extraction and real-time RT-PCR.

RNA was isolated using TRIzol (Invitrogen, catalog T9424) according to the manufacturer’s protocol and quantified with a NanoDrop 3300 fluorospectrometer. One microgram of total RNA was reverse transcribed into cDNA using an iScript cDNA synthesis kit (Bio-Rad). Real-time PCR amplification of target genes was performed using iTaq Universal SYBR green Supermix (Bio-Rad) and specific primers (Supplemental Table 3) on an AB 7900HT instrument (Applied Biosystems) with the following settings: 94°C for 10 minutes, 40 cycles of amplification at 94°C for 15 seconds and 60°C for 1 minute, and an extension step of 7 minutes at 72°C. Relative mRNA levels were calculated with the ΔΔCt method using GAPDH for standardization. Results are presented as mean ± SD of 3 biological replicates per experimental group.

### RNA-seq and pathway analysis.

RNA-seq libraries were prepared from 1 μg of total RNA that was extracted as described above. mRNA libraries were generated using a NEBNext Ultra II RNA library prep kit from Illumina (New England Biolabs, Inc.), sequenced, and analyzed to identify DEGs as previously described (70, 71). Data were deposited in the NCBI GEO database (GSE271466; token: ihgxgoaqhrkhrkf). Pathway and regulator analyses were performed using Metascape (72) and Ingenuity Pathway Analysis (IPA, Qiagen).

### RPPA.

OVCAR5 cells were cocultured with fully matured VNPADs for 3 days, as described above. Cells were subsequently isolated by FACS in the GFP channel and cell pellets were precipitated by centrifuging at 1000 rpm for 3 minutes. Cell pellets were flash frozen and processed for protein extraction and RPPA analysis at the MD Anderson Cancer Center Proteomics Core Facility. Broadly, protein extracts (*n* = 3 replicates per group) were diluted 2-fold and spotted onto nitrocellulose-coated slides. The proteins were detected by probing with specific antibodies (501 antibodies) and the signal was amplified using a tyramide amplification system and visualized via DAB reaction. Digital images of the slides were obtained by scanning (Huron TissueScope scanner). Mean net intensities of spots were used for curve fitting for each slide. Relative protein levels were determined by using RPPASPACE (https://bioinformatics.mdanderson.org/public-software/rppaspace/).

### Western blotting.

Protein lysates were prepared using RIPA buffer and quantified using Bradford reagent. Proteins were resolved by PAGE and then transferred onto a PVDF membrane using a wet electrophoresis system. Membranes were blocked in TBS-Tween containing 5% BSA for 1 hour and then incubated with primary antibodies (1:1000 dilution, overnight at 4°C) followed by secondary antibody (anti-rabbit/mouse–horseradish peroxidase 1:1000 dilution) for 1 hour at room temperature. Generation and detection of chemiluminescence signal used SuperSignal West Pico PLUS (Thermo Fisher Scientific, catalog 34580) and an ImageQuant LAS 4000 imaging system. Membranes were treated with Restore Western Blot Stripping Buffer (Thermo Fisher Scientific, catalog 21059), blocked, and then incubated with primary antibody to detect additional proteins. The antibodies are listed in Supplemental Table 2.

### Quantification of lipid accumulation.

The uptake or generation of lipids in vitro was measured with AdipoRed (Lonza, catalog PT-7009) as described by the manufacturer. Briefly, cells were seeded on 96-well plates and evaluated at 80% confluence. For this, media were removed, cells were rinsed twice with PBS, and then treated with 5 μL of AdipoRed mixed in 200 μL of PBS for 10 minutes under culture conditions. Fluorescence was measured with a Spectramax Gemini XS reader set at 485 nm and 572 nm for excitation and emission wavelengths, respectively. Data was analyzed and plotted using GraphPad Prism 6.

### ELISA.

Following coculture of OC cells with adipocytes, cells were separated by FACS. Both cocultured sorted and control cells were seeded on 6-cm dishes for 24 hours in serum-free media before conditioned media were collected and used for ELISA. Complement C3, C3a, and C5 were measured using ELISA kits (catalog ab108823m, ab133037, and ab125963 for C3, C3a, and C5, respectively; Abcam) by using conditioned media and ascites specimens, processed as described above. All measurements were carried out in duplicate and averaged.

### Colony formation assay.

Colony formation was evaluated by seeding 500 cells into 6-well high-adhesion plates (Corning). Cells were grown in 3 mL of RPMI 1640 medium (Invitrogen), which was supplemented with 10% FBS, 1% GlutaMax (Thermo Fisher Scientific), and 1% penicillin/streptomycin (Corning). Cells were cultured for 4 weeks, and media were refreshed every 4 days. At the endpoint of the experiments, cells were washed with PBS and fixed with 4% paraformaldehyde (Electron Microscopy Sciences) for 30 minutes and then stained with crystal violet for 5 minutes (0.025% w/v, Sigma-Aldrich). Colony numbers were counted in each well, excluding small diameter (less than 50 cells) colonies.

### Migration assay.

Cell migration was assessed by using Boyden chambers with a pore size of 8 μm. Cells suspended in 200 μL serum-free media were seeded in the upper well of the chamber (75,000 cells/well). Media in the lower chamber were supplemented with 15% serum. Cells were allowed to migrate for 12 hours and were then fixed with 4% paraformaldehyde. The filters were removed, washed 3 times with PBS, and stained with crystal violet for visualization. Images were captured using a digital camera attached to a light microscope and colonies counted using the Fiji software.

### In vivo xenograft experiments.

Experiments were performed with female, 6–7 weeks old, athymic nude mice (*Foxn1^nu^*) purchased from Envigo. Mice were injected i.p. with 2 million OVCAR5 cells maintained in monoculture or FACS isolated after coculture with adipocytes. In another experiment, mice were injected i.p. with 1 million OVCAR5 cells stably transduced with shRNA targeting C3 (shC3) or control shRNA (shCtrl). Mice were euthanized 4 weeks later to evaluate tumor burden. Tumor weights and volumes, numbers of tumor greater than 1 mm, and ascites volumes were recorded. Tumor length (*l*), width (*w*), and height (*h*) were determined with digital calipers and tumor volumes (*v*) were calculated with the formula *v* = ½ × *l* × *w* × *h*. In another experiment, C57BL/6 mice were purchased from Envigo and were injected i.p. with 5 million ID8 cells stably transduced with shCtrl or shC3. Mice were euthanized after 4 weeks to evaluate tumor burden as described above.

### Statistics.

Data are presented as mean ± SD with associated *P* values. Statistical analysis was performed by using a 2-tailed Student’s *t* test, 1-way ANOVA, or 2-way ANOVA (GraphPad Prism 6 software). A *P* value of less than 0.05 was considered statistically significant.

### Study approvals.

All human specimens utilized in these studies were de-identified and obtained under IRB-approved protocols. The fresh tumor and ascites specimens were collected from consenting individuals under Northwestern University’s IRB-approved protocol STU00202468. Paraffin-embedded HGSOC specimens were acquired under IRB-approved protocol STU00217148. The research biobanking protocol at UC-Christus Health Network, Santiago, Chile was approved by the IRB and all recruited patients signed informed consent under protocol FONDECYT 1201083. Animal studies were conducted according to a protocol approved by the Institutional Animal Care and Use Committee of Northwestern University (no. IS00023799).

### Data availability.

All raw data can be found in the Supporting Data Values files. All other data can be made available upon request. RNA-seq analysis results are deposited in the NCBI GEO database (GSE271466; token: ihgxgoaqhrkhrkf).

## Author contributions

AV and DM conceived and designed the research. AV, AMI, GZ, YZ, HH, SK, FGV, and FG performed experiments. AV, AMI, GZ, YZ, MCF, SK, and FGV analyzed data. AV, AMI, HC, JJW, MCF, AK, and DM interpreted results of experiments. AV and AMI prepared figures. AV and DM drafted the manuscript, which was edited and revised by AV, AMI, HC, JJW, MCF, AK, and DM. JJW, FG, AK, and MCF provided key materials for study. DM and AV provided funding for the study.

## Supplementary Material

Supplemental data

Unedited blot and gel images

Supporting data values

## Version 1 02/18/2025

In-Press Preview

## Version 2 03/24/2025

Electronic publication
