# Supplementary material for: Complement activation at the interface between adipocytes and cancer cells drives tumor progression
Source: JCI Insight. 2025 Feb 18;10(6):e184935. doi: 10.1172/jci.insight.184935 (PMC11949041; doi:10.1172/jci.insight.184935)
Supplement: Supplemental data [file jciinsight-10-184935-s130.pdf]

Complement activation at the interface between adipocytes and cancer cells drives tumor progression

Supplemental Material

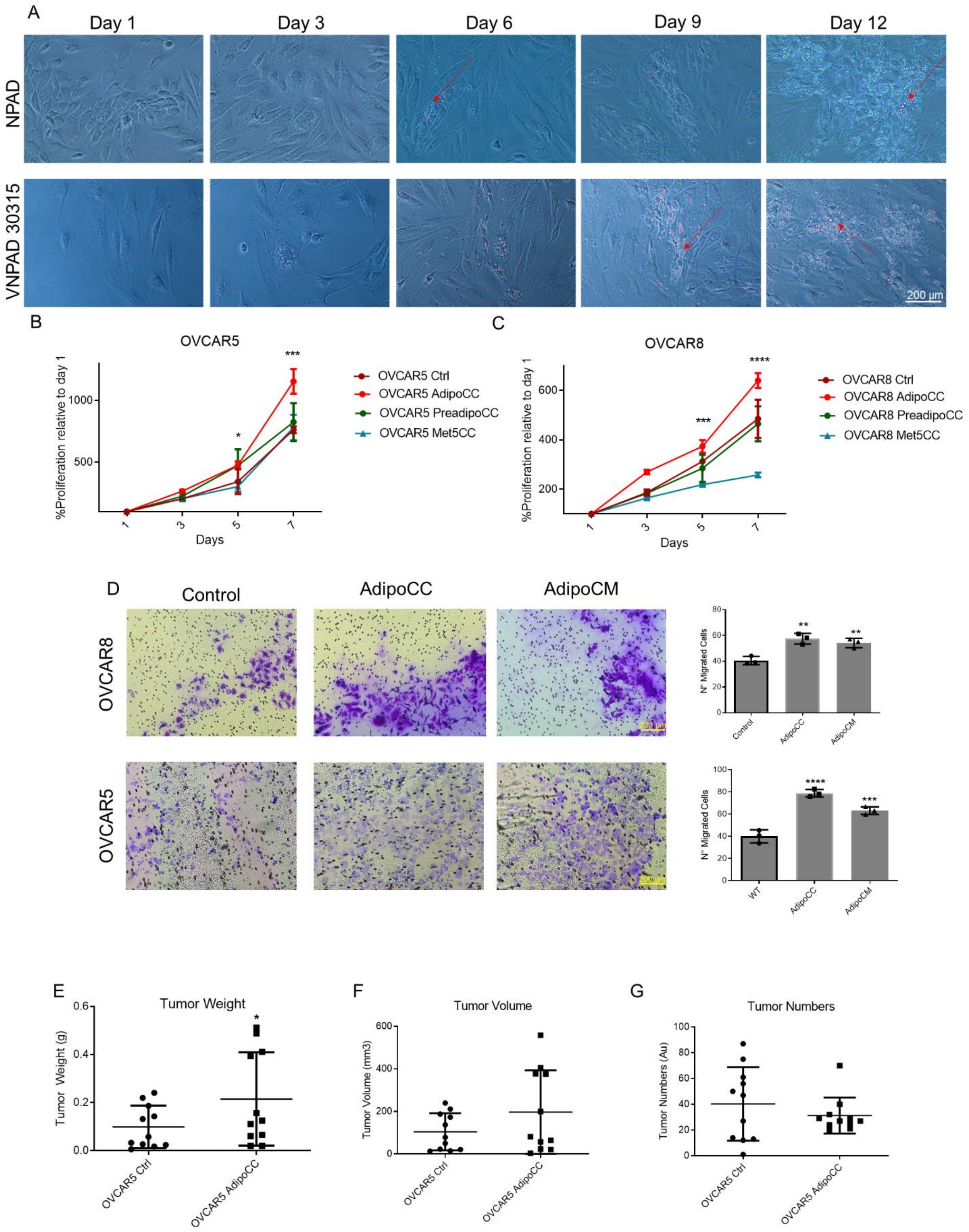
A
Day 1
Day 3
Day 6
Day 9
Day 12
NPAD
VNPAD 30315
200 µm
B
OVCAR5
%Proliferation relative to day 1
Days
OVCAR5 Ctrl
OVCAR5 AdipoCC
OVCAR5 PreadipoCC
OVCAR5 Met5CC
C
OVCAR8
%Proliferation relative to day 1
Days
OVCAR8 Ctrl
OVCAR8 AdipoCC
OVCAR8 PreadipoCC
OVCAR8 Met5CC
D
Control
AdipoCC
AdipoCM
OVCAR8
OVCAR5
N° Migrated Cells
Control
AdipoCC
AdipoCM
WT
E
Tumor Weight
Tumor Weight (g)
OVCAR5 Ctrl
OVCAR5 AdipoCC
F
Tumor Volume
Tumor Volume (mm3)
OVCAR5 Ctrl
OVCAR5 AdipoCC
G
Tumor Numbers
Tumor Numbers (Au)
OVCAR5 Ctrl
OVCAR5 AdipoCC

Supplemental Figure S1: Characterization of an *in-vitro* adipocyte co-culture model. (A) Representative images of differentiation over a period of 12 days of adipocytes cell lines NPAD and VNPAD 30315. Red arrows indicate lipid droplets stained with AdipoRed. (**B-C**) Proliferation assay (mean ± SD, n=3) of OVCAR5 **(B)** and OVCAR8 **(C)** cells in monoculture (Ctrl), co-culture with mature adipocytes (AdipoCC), with undifferentiated adipocytes (PreadipoCC) and with mesothelial cells (MeT5CC). Cells were directly co-cultured for 3 days, separated by FACS before proliferation assessment using CCK8 assay. **(D)** Transwell migration assay of OVCAR5 and OVCAR8 cells in monoculture, co-culture with adipocytes or after treatment with VNPAD conditioned media (mean ± SD, n=3). **(E-G)** Total tumor weight **(E)**, total tumor volume **(F)** and numbers of tumors **(G)** in nude female mice injected IP with OVCAR5 cells maintained as monoculture or co-cultured for 3 days with adipocytes (means ± SD, n=11 per group). For all panels, *p<0.05; **p<0.01; ***p<0.001; ****p<0.0001. 2-way ANOVA was used to analyze figure B and C. 1-way ANOVA was use to analyze figure D. Unpaired 2-tailed t test was performed to analyze figure E-G.

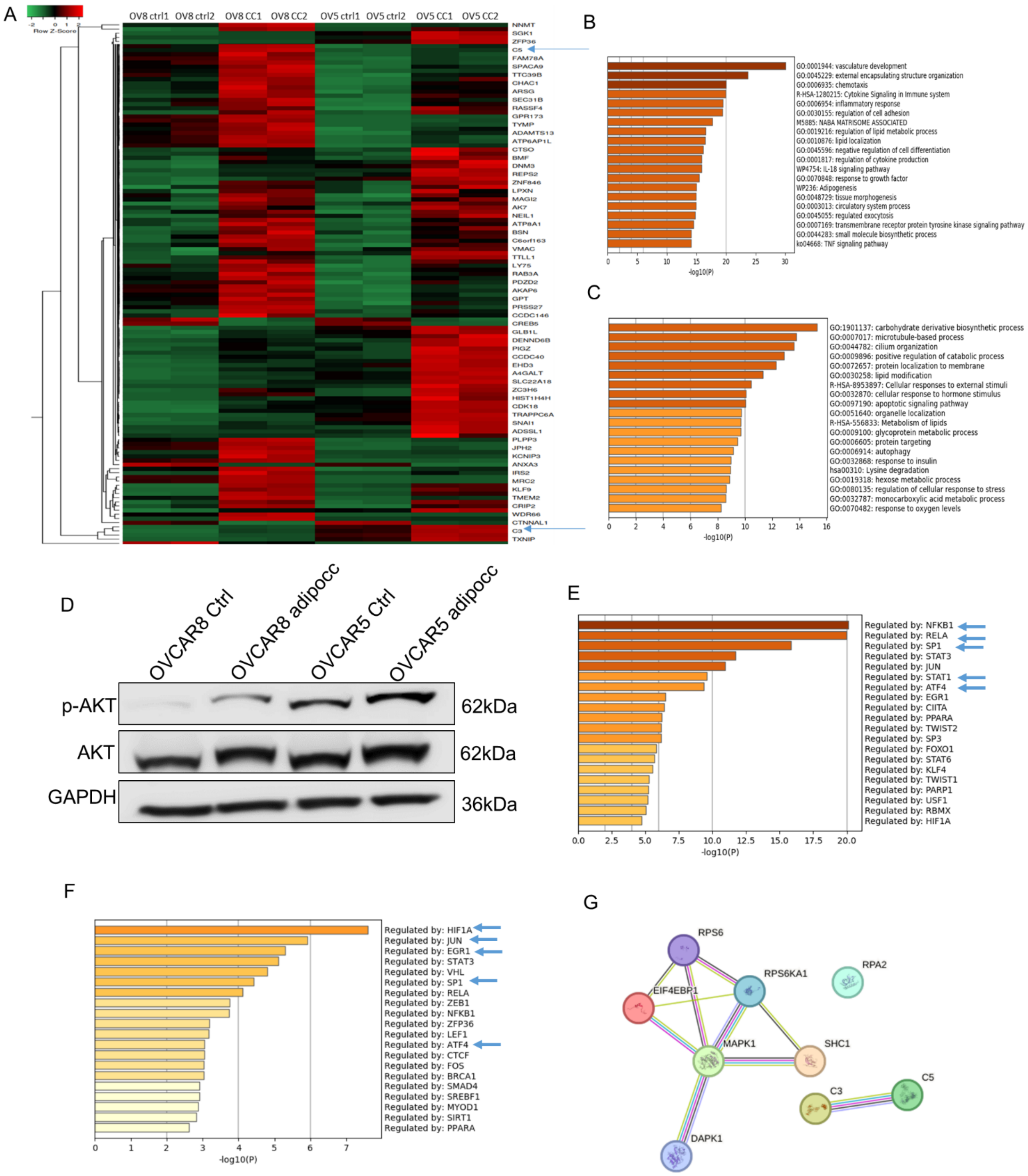
A
-2 0 1 2
Row Z-Score
OV8 ctrl1 OV8 ctrl2 OV8 CC1 OV8 CC2 OV5 ctrl1 OV5 ctrl2 OV5 CC1 OV5 CC2
NNMT
SGK1
ZFP36
C5
FAM78A
SPACA9
TTC39B
CHAC1
ARSG
SEC31B
RASSF4
GPR173
TYMP
ADAMTS13
ATP6AP1L
CTSO
BMF
DNM3
REPS2
ZNF846
LPXN
MAGI2
AK7
NEIL1
ATP8A1
BSN
C6orf163
VMAC
TTLL1
LY75
RAB3A
PDZD2
AKAP6
GPT
PRSS27
CCDC146
CREB5
GLB1L
DENND6B
PIGZ
CCDC40
EHD3
A4GALT
SLC22A18
ZC3H6
HIST1H4H
CDK18
TRAPPC6A
SNAI1
ADSSL1
PLPP3
JPH2
KCNIP3
ANXA3
IRS2
MRC2
KLF9
TMEM2
CRIP2
WDR66
CTNNAL1
C3
TXNIP
B
GO:0001944: vasculature development
GO:0045229: external encapsulating structure organization
GO:0006935: chemotaxis
R-HSA-1280215: Cytokine Signaling in Immune system
GO:0006954: inflammatory response
GO:0030155: regulation of cell adhesion
M5885: NABA MATRISOME ASSOCIATED
GO:0019216: regulation of lipid metabolic process
GO:0010876: lipid localization
GO:0045596: negative regulation of cell differentiation
GO:0001817: regulation of cytokine production
WP4754: IL-18 signaling pathway
GO:0070848: response to growth factor
WP236: Adipogenesis
GO:0048729: tissue morphogenesis
GO:0003013: circulatory system process
GO:0045055: regulated exocytosis
GO:0007169: transmembrane receptor protein tyrosine kinase signaling pathway
GO:0044283: small molecule biosynthetic process
ko04668: TNF signaling pathway
0 5 10 15 20 25 30
-log10(P)
C
GO:1901137: carbohydrate derivative biosynthetic process
GO:0007017: microtubule-based process
GO:0044782: cilium organization
GO:0009896: positive regulation of catabolic process
GO:0072657: protein localization to membrane
GO:0030258: lipid modification
R-HSA-8953897: Cellular responses to external stimuli
GO:0032870: cellular response to hormone stimulus
GO:0097190: apoptotic signaling pathway
GO:0051640: organelle localization
R-HSA-556833: Metabolism of lipids
GO:0009100: glycoprotein metabolic process
GO:0006605: protein targeting
GO:0006914: autophagy
GO:0032868: response to insulin
hsa00310: Lysine degradation
GO:0019318: hexose metabolic process
GO:0080135: regulation of cellular response to stress
GO:0032787: monocarboxylic acid metabolic process
GO:0070482: response to oxygen levels
0 2 4 6 8 10 12 14 16
-log10(P)
D
OVCAR8 Ctrl
OVCAR8 adipocc
OVCAR5 Ctrl
OVCAR5 adipocc
p-AKT 62kDa
AKT 62kDa
GAPDH 36kDa
E
Regulated by: NFKB1
Regulated by: RELA
Regulated by: SP1
Regulated by: STAT3
Regulated by: JUN
Regulated by: STAT1
Regulated by: ATF4
Regulated by: EGR1
Regulated by: CIITA
Regulated by: PPARA
Regulated by: TWIST2
Regulated by: SP3
Regulated by: FOXO1
Regulated by: STAT6
Regulated by: KLF4
Regulated by: TWIST1
Regulated by: PARP1
Regulated by: USF1
Regulated by: RBMX
Regulated by: HIF1A
0.0 2.5 5.0 7.5 10.0 12.5 15.0 17.5 20.0
-log10(P)
F
Regulated by: HIF1A
Regulated by: JUN
Regulated by: EGR1
Regulated by: STAT3
Regulated by: VHL
Regulated by: SP1
Regulated by: RELA
Regulated by: ZEB1
Regulated by: NFKB1
Regulated by: ZFP36
Regulated by: LEF1
Regulated by: ATF4
Regulated by: CTCF
Regulated by: FOS
Regulated by: BRCA1
Regulated by: SMAD4
Regulated by: SREBF1
Regulated by: MYOD1
Regulated by: SIRT1
Regulated by: PPARA
0 1 2 3 4 5 6 7
-log10(P)
G
RPS6
RPS6KA1
RPA2
EIF4EBP1
MAPK1
SHC1
C3
C5
DAPK1

Supplemental Figure S2. RNA-Seq analysis comparing DEGs and enriched pathways of OVCAR5 and OVCAR8 cells mono and co-cultured with adipocytes. Pathway analysis of RNA-sequencing data was performed by using the web-based Metascape portal. **(A)** Heatmap showing the top common DEGs (FDR<0.05) identified through RNA-sequencing analysis in OVCAR8 and OVCAR5 comparing co-culture with adipocytes vs mono-culture. C3 and C5 are indicated with blue arrows. **(B)** Pathway enrichment analysis of DEGs (FDR<0.05) identified in OVCAR5 cells co-cultured with adipocytes. **(C)** Pathway enrichment analysis of DEGs (FDR<0.05) identified in OVCAR8 cells co-cultured with adipocytes. **(D)** Western blot analysis for AKT phosphorylation in OVCAR8 and OVCAR5 cells mono and co-cultured with adipocytes. **(E)** Main effectors for transcriptomic changes induced by co-culture with adipocytes in OVCAR5 cells. **(F)** Main effectors for transcriptomic changes induced by co-culture with adipocytes in OVCAR8 cells. (**G**) String analysis of protein-to-protein interactions among downregulated proteins identified by RPPA. C3 and C5 were added to the analysis.

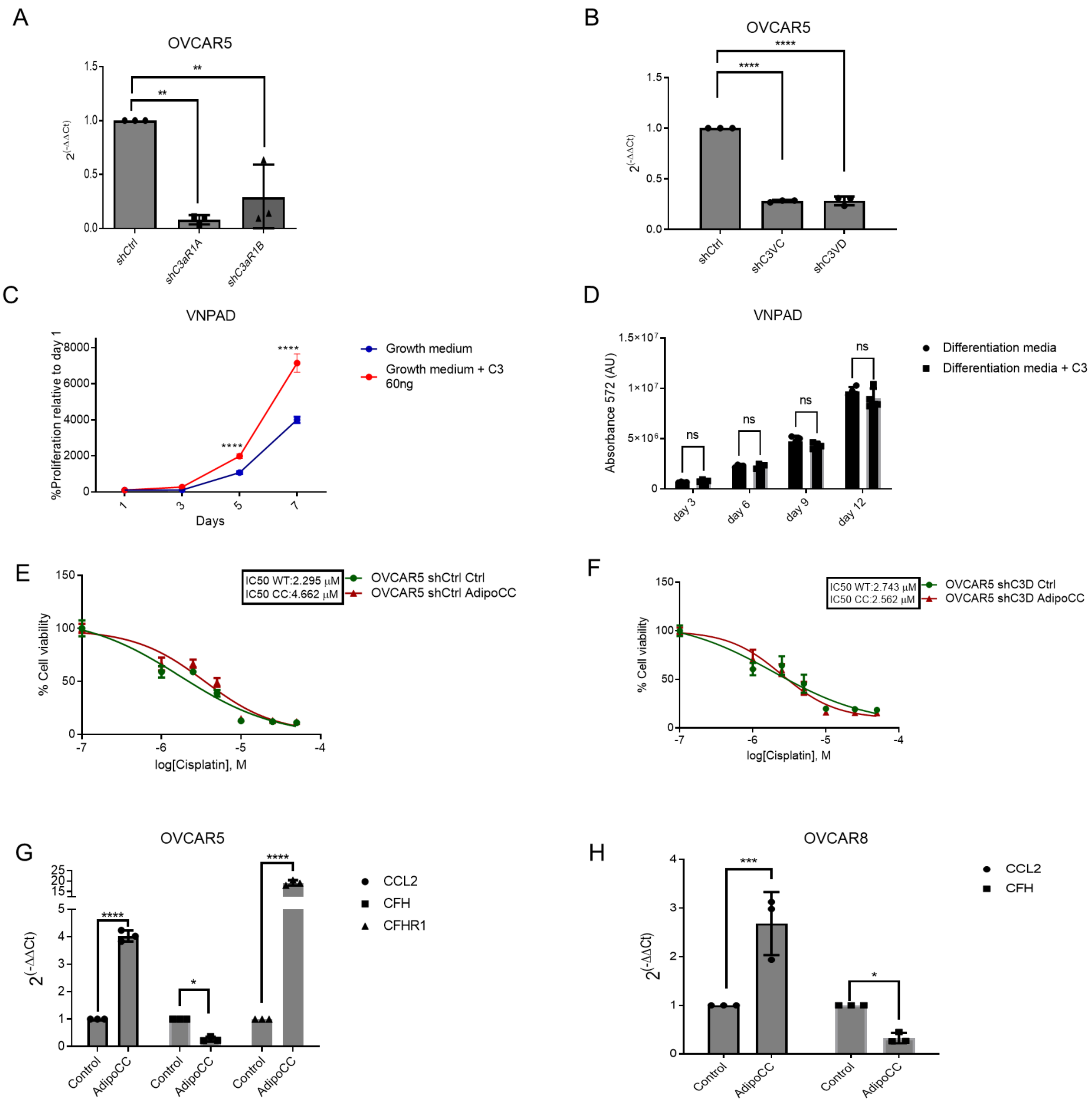
A
OVCAR5
2(-ΔΔCt)
shCtrl
shC3aR1A
shC3aR1B
**
B
OVCAR5
2(-ΔΔCt)
shCtrl
shC3VC
shC3VD
****
C
VNPAD
%Proliferation relative to day 1
Days
Growth medium
Growth medium + C3 60ng
****
D
VNPAD
Absorbance 572 (AU)
day 3
day 6
day 9
day 12
ns
Differentiation media
Differentiation media + C3
E
% Cell viability
log[Cisplatin], M
IC50 WT:2.295 µM
IC50 CC:4.662 µM
OVCAR5 shCtrl Ctrl
OVCAR5 shCtrl AdipoCC
F
% Cell viability
log[Cisplatin], M
IC50 WT:2.743 µM
IC50 CC:2.562 µM
OVCAR5 shC3D Ctrl
OVCAR5 shC3D AdipoCC
G
OVCAR5
2(-ΔΔCt)
Control
AdipoCC
CCL2
CFH
CFHR1
****
*
H
OVCAR8
2(-ΔΔCt)
Control
AdipoCC
CCL2
CFH
***
*

Supplemental Figure S3. C3 and its regulators are affected by co-culture with adipocytes. (**A**) qRT-PCR measurements of C3AR1 mRNA in OVCAR5 cells transduced with shRNA targeting C3AR1 (shC3AR1 A, shC3AR1 B) or non-targeting shRNA (shCtrl) (means ± SD, n=3). (**B**) qRT-PCR measurements of C3 mRNA in OVCAR5 cells transduced with shRNA targeting C3 (shC3C, shC3D) or non-targeting shRNA (shCtrl) (means ± SD, n=3). (**C**) Proliferation (CCK8 assay) of pre-adipocyte VNPAD cells cultured in the base medium vs. treated with 50 ng recombinant C3 (means ± SD, n=4). (D) Lipid accumulation measured with AdipoRed on days 3, 6, 9 and 12 post-differentiation in VNPAD cells cultured in differentiation media with or without 50 ng recombinant C3 (means ± SD, n=4) **(E-F)** Effects of co-culture with adipocytes on responsiveness to platinum in OVCAR5 control (shCtrl, **E**) and C3 know-down cells (shC3D, **F**). IC_{50} to cisplatin is indicated for each graph. (**G, H**) qPCR analysis of complement pathway activation regulators CCL2, CFH and CFHR1 in OVCAR5 (**C**) and OVCAR8 (**D**) cells co-cultured with adipocyte vs monocultured (control). Values in all panels are means ± SD. *p<0.05; **p<0.01; ***p<0.001; ****p<0.0001. 2-way ANOVA was used to analyze figure C. 1-way ANOVA was use to analyze figure A, B, D, G and H.

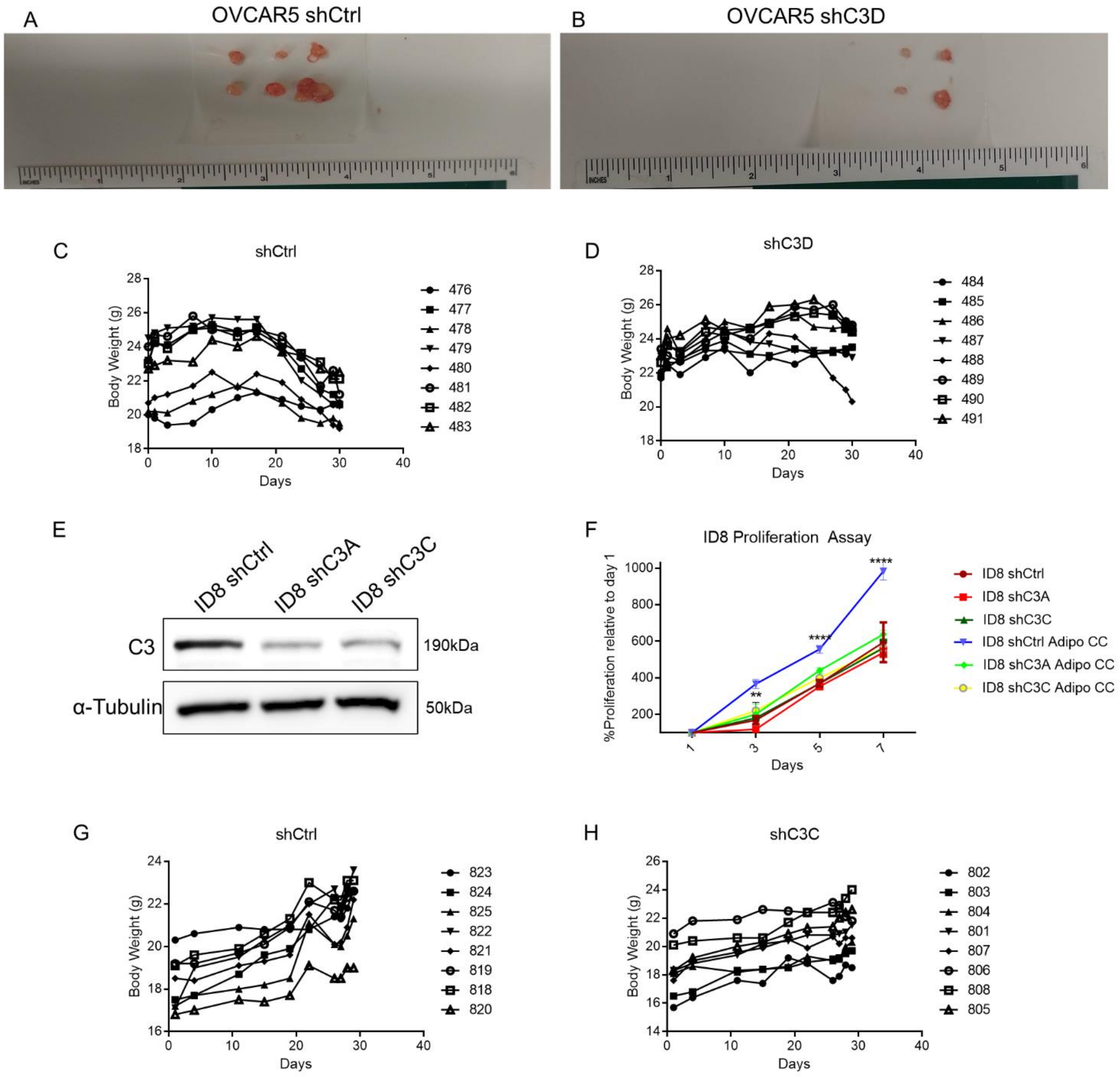
A
OVCAR5 shCtrl
B
OVCAR5 shC3D
C
shCtrl
Body Weight (g)
Days
476
477
478
479
480
481
482
483
D
shC3D
Body Weight (g)
Days
484
485
486
487
488
489
490
491
E
ID8 shCtrl
ID8 shC3A
ID8 shC3C
C3
190kDa
α-Tubulin
50kDa
F
ID8 Proliferation Assay
%Proliferation relative to day 1
Days
ID8 shCtrl
ID8 shC3A
ID8 shC3C
ID8 shCtrl Adipo CC
ID8 shC3A Adipo CC
ID8 shC3C Adipo CC
G
shCtrl
Body Weight (g)
Days
823
824
825
822
821
819
818
820
H
shC3C
Body Weight (g)
Days
802
803
804
801
807
806
808
805

Supplemental Figure S4. C3 knock down effects in *in-vivo* mouse models. Representative images of tumors formed by OVCAR5 shCtrl (**A**) and shC3D (**B**) 4 weeks after IP injection into nude mice. Weights of mice injected with OVCAR5 shCtrl (**C**) and shC3D (**D**) during the duration of the experiment. (**E**) Western blot analysis for C3 in ID8 cells stably transfected with shRNA targeting C3 (two clones, shC3A and shC3C) or control shRNA (shCtrl). (**F**) Proliferation (CCK8 assay) of shCtrl and shC3 (A and C) ID8 cells maintained as monoculture (Ctrl) vs. co-cultured with adipocytes (AdipoCC) (values are means ± SD, n=3). Weights of mice injected with ID8 shctrl (**G**) and shC3C (**H**) during the duration of the experiment. Values in all panels are means ± SD. *$p<0.05$; **$p<0.01$; ***$p<0.001$; ****$p<0.0001$. 2-way ANOVA was used to analyze figure F.

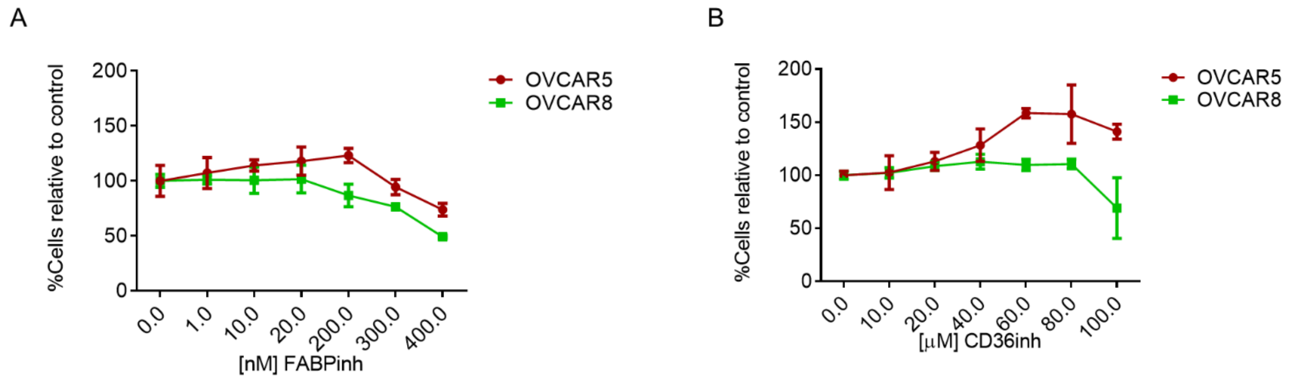

Supplemental Figure S5. Effects of FABP and CD36 inhibitors on cell viability. Effects of FAPB **(A)** and CD36 **(B)** inhibitors on viability of OVCAR5 and OVCAR8 cells measured as relative cell viability. Cells were treated with a dose range of inhibitors for 7 days and viable cells were measured by using the CCK8 assay.

Supplemental Table S1: Culture media.

**Cell line**	**Cell Culture Media**
**OVCAR5**	RPMI 1640 (Corning), 10% fetal bovine serum (FBS) (Corning), 1% penicillin/streptomycin (Sigma), and 1X GlutaMAX (Gibco).
**OVCAR8, MET5A and ID8**	DMEM (Corning), 10% fetal bovine serum (FBS) (Corning), 1% penicillin/streptomycin (Sigma).
**VNPAD 30319 and NPAD**	**Growing media:** Human Adipocyte & Preadipocyte Media (Cell Applications), 10% fetal bovine serum (FBS) (Corning), 1% penicillin/streptomycin (Sigma); **Differentiation media:** Human Adipocyte Differentiation Medium (Cell Applications).
**Primary Cells**	DMGM: MCDB 105 medium powder (Sigma Aldrich) + Medium 199 (Corning), 10% fetal bovine serum (FBS) (Corning), 1% penicillin/streptomycin (Sigma).

Supplemental Table S2: List of primary antibodies.

**Antibodies**	**Source**	**Cat#**
C3	Invitrogen	PA5-21349
AKT	Cell Signaling Technology	9272S
Phospho-AKT (Ser473)	Cell Signaling Technology	9271S
ATF4	Cell Signaling Technology	97038S
CD3	Invitrogen	MA5-14524
elF2α	Cell Signaling Technology	9722S
Phospho-elF2α (S51)	Cell Signaling Technology	3597S
GAPDH	Thermo Scientific	AM4300
α-Tubulin	Proteintech	66031-1-1g

Supplemental Table S3 List of primers for quantitative PCR.

Gene	Forward Primer (5' to 3')	Reverse Primer (5' to 3')
C3	AGCAGCTGGCCTTCAGACAA	GACTTGGGAGTCGATGGCGA
C5	GGGAAAACCTGGGGACAGGA	TTTTCAGATGCTCCAACACGGA
CCL2	ATAGCAGCCACCTTCATTCCCC	CACAGATCTCCTTGGCCACAA
CFH	ACGTTGTGAACAGAGTTAGCTGG	TAGCCTGGGTGCCTTCTGGA
CFR1	TCATGTGTAGAACGGGGCTGG	GGGCGGATTCACACAGGAAG
ATF4	GCGGGTTTTGGATTGGTGGG	CCCGACTACGCTTTCCCGAT
C3AR1	CCTGCTGATGTGGTCTCACCTA	CCTTGTGGTAGCTCAGACTCGT

Supplemental Table S4: Top 100 DEGs in OVCA5 cells co-cultured with adipocytes vs monoculture.

Hgnc symbol	LogFC	p-Value
COL1A2	9.161625161	6.66864E-22
COL3A1	8.366298245	1.08381E-42
ASB4	7.896328608	8.38071E-17
PTGIS	7.744664782	4.01529E-15
COL5A1	6.255225206	8.88984E-41
FIBIN	6.229667025	2.95101E-27
MMP2	6.008467014	1.16344E-19
HP	5.708228544	6.8271E-260
ADH1B	5.609395473	1.15775E-15
NID1	5.503511705	8.99497E-25
PXDN	5.48703416	1.3828E-14
ISLR	5.24107502	1.41028E-12
TRPM8	5.210358434	2.53458E-12
AL592158.1	5.138947211	1.12826E-30
NLRP6	5.050966659	6.90452E-18
KLRD1	5.01018195	2.61916E-15
FBLN2	4.91063857	9.14607E-16
KCNB1	4.888849607	4.99893E-12
CD248	4.710432472	5.29781E-14
EFEMP1	4.612665046	4.96016E-13
RNF112	4.587245705	3.5086E-13
DCN	4.567801361	3.87031E-26
CXCL8	4.555262517	7.9965E-102
NDUFA4L2	4.519478146	1.99377E-07
VCAN	4.499923793	3.57367E-16
TMEM100	4.498935741	2.26885E-14
C10orf10	4.496392082	1.62947E-36
IGFBP5	4.394500105	8.47053E-05
ADAMTS2	4.294895711	7.19653E-14
COL6A3	4.264723892	7.18968E-51
NDRG1	4.251325939	3.04134E-12
COL4A1	4.216081932	7.27509E-31
MMP7	4.147451092	2.17578E-19
ENPP2	4.119303837	3.51167E-74
ROS1	4.041791289	1.74986E-21
CA9	4.015163041	9.56504E-09
CP	3.934517608	3.00059E-36
NR4A1	3.890917872	1.0556E-238
CHRD	3.886507953	6.33751E-67

BGN	3.872531398	2.06067E-15
SLC2A3	3.797467235	1.44985E-06
SERPINA1	3.791962398	1.04155E-20
RIPOR2	3.783991345	9.46189E-22
GALNT15	3.749487814	2.30008E-14
CD36	3.690359257	1.79129E-38
STC1	3.546012292	1.02096E-06
IFITM1	3.512553685	2.8815E-112
IL6	3.512271902	4.01278E-22
LGALS12	3.488796574	3.77302E-31
SERPINA5	3.483331383	3.2665E-125
HIF3A	3.432137187	3.19906E-24
EGR2	3.429494259	2.32862E-29
C6orf223	3.428972222	6.02146E-17
C4A	3.369290047	4.76113E-15
MAFB	3.349206887	1.96498E-22
ANPEP	3.332712403	2.66712E-34
WNT6	3.320628867	8.02157E-15
PLIN2	3.300903675	2.03736E-18
TIE1	3.283974388	2.33495E-14
AGT	3.249649824	1.48925E-18
AC105942.1	3.235894517	6.28548E-11
DAAM2	3.218636353	3.1804E-288
C4B	3.213876393	7.44727E-25
NLRP1	3.200835218	1.32314E-11
TP63	3.185239033	1.03387E-26
STAC2	3.162615069	3.93173E-28
PGC	3.157252218	1.57271E-13
ALOX15B	3.122689113	3.0538E-15
CD14	3.119489189	1.85596E-70
SDS	3.119145578	1.1645E-13
ZNF467	3.108713033	6.63886E-44
FOXJ1	3.108067811	1.60473E-20
ABCA6	3.086623142	1.89668E-51
SERPING1	3.085545734	1.48771E-31
NOX5	3.082321785	1.30121E-09
LINC02015	2.975166391	6.21389E-09
SYNPO2	2.973299868	5.03847E-32
CRYM	2.967734539	1.36528E-21
WFDC3	2.96121562	7.76764E-19
NFATC4	2.956100141	1.67408E-61
LOX	2.93963806	6.65932E-06
COL1A1	2.919511581	1.484E-31

LINC02577	2.915943043	1.91578E-17
PCSK9	2.911231184	5.7508E-46
COL4A2	2.904362761	3.54962E-22
NR4A2	2.854585638	9.72419E-60
KCNJ2-AS1	2.835149551	3.03206E-12
TOGARAM2	2.827125591	1.1046E-27
NUPR1	2.818053479	1.73488E-08
C3	2.815643193	3.58622E-54
EDN2	2.799702947	2.12454E-32
C1S	2.797940988	4.25062E-45
FBN1	2.79328653	6.1277E-20
MYCBPAP	2.787457256	8.20517E-15
SULT1B1	2.783917331	7.64691E-13
LRP1	2.754090248	2.45711E-57
CDK5R2	2.741579578	9.50795E-10
CYTH4	2.715529425	2.40234E-19
ACKR3	2.69191749	4.22867E-09
PTGS2	2.670512917	8.87918E-24

Supplemental Table S5: Top 100 DEGs in OVCA8 cells co-cultured with adipocytes vs monoculture.

Hgnc symbol	LogFC	p-Value
CDH16	9.027408354	1.361E-129
DMBT1	7.631959794	4.7519E-156
SAA1	7.40654607	7.6066E-116
GGT5	5.557170601	1.57475E-15
TNS4	5.265489719	5.65291E-77
COL15A1	5.103111515	2.23717E-77
FPR1	4.827072621	6.16383E-16
CRISPLD2	4.798634597	1.57475E-15
TSC22D3	4.642613793	5.65291E-77
PPARGC1A	4.608447174	2.83168E-69
NOG	4.47475249	2.0073E-153
HTRA1	4.471593633	4.289E-261
SCNN1A	4.427359952	1.7707E-215
ADAMTS14	4.408234961	4.62879E-82
DHRS3	4.181607939	1.017E-140
KRT81	4.056710105	9.32317E-97
RAB3C	4.003097207	3.84024E-12
C1orf186	3.954391274	1.27717E-14
ABCC3	3.927116018	1.0222E-112
C3	3.74559042	5.3534E-17
DDIT4	3.688730232	2.1285E-202
ITGB4	3.615538684	1.24212E-12
SUSD2	3.597530628	3.41706E-34
XKR4	3.590653209	5.35348E-11
LRRC4	3.534349543	1.63772E-10
PTGES	3.414046352	3.4101E-195
SLPI	3.408751979	8.91005E-37
RRAD	3.407211162	6.1473E-142
EDN2	3.267634085	1.33309E-29
MYLK3	3.23736571	8.11204E-40
SLC26A2	3.20937972	1.97626258336499e-323
NPR3	3.203405425	6.9677E-191
HIF3A	3.192445777	2.01607E-17
PDE3A	3.168717482	1.67291E-17
CU639417.1	3.056189702	1.107E-99
ANGPTL4	3.043921657	1.11962E-43
KRT86	3.036106171	4.77396E-30
AP3B2	2.989700841	6.43497E-14
CCL20	2.912913723	4.7294E-22

COL5A1	2.905043146	6.62955E-23
SLC1A3	2.792661991	3.0221E-124
DGKI	2.761966529	2.23323E-08
PER1	2.724908732	1.6088E-122
PDE6A	2.712536108	4.68165E-32
CPEB4	2.703207967	1.7469E-159
MAP2	2.698794778	2.48398E-14
EFEMP1	2.696149035	3.4682E-212
IGFBP4	2.6895405	7.8073E-248
KCNG2	2.663905521	2.50892E-10
KLF9	2.662511354	6.6295E-197
STRA6	2.653400707	1.66368E-17
SAMD11	2.534545438	2.5925E-18
TGFBR1	2.534524365	3.5817E-199
PDE4D	2.53015681	2.3362E-119
BEST1	2.515333181	5.45411E-32
HHIPL2	2.51090798	7.97984E-07
PRLR	2.505229715	1.68578E-09
IRS2	2.48404054	1.6628E-179
NRCAM	2.42640221	8.97371E-25
DEPTOR	2.407410795	6.23925E-76
FKBP5	2.399014131	2.0177E-181
SMAD6	2.323832024	1.12131E-79
AREG	2.29121023	3.42869E-17
NUPR1	2.289873259	4.33987E-07
ID3	2.27915456	7.8043E-115
SHISA9	2.27709137	6.2902E-154
TFCP2L1	2.276662848	3.77707E-45
ASS1	2.273483257	3.3649E-119
MT1X	2.243526947	1.0746E-127
CEBPD	2.242577945	3.7329E-154
DNAH7	2.23850191	1.88194E-07
ALPP	2.216244569	4.74712E-21
PLEKHG6	2.19373456	1.42224E-07
BIRC3	2.156220128	2.64672E-46
CNR1	2.109841865	1.9262E-11
GCNT1	2.109756187	5.6153E-105
NEBL	2.083268807	5.07726E-19
IL1R1	2.070342888	1.40925E-48
KCNIP3	2.067269518	2.91063E-98
PRODH	2.062078691	1.64601E-15
SLC16A12	2.061983968	4.91542E-14
GDF6	2.056596289	1.6776E-125

PTK2B	2.055275288	2.2381E-79
EPAS1	2.037122097	1.383E-128
THRB	2.030996559	5.18586E-27
CHAD	2.029531337	8.16634E-07
RASD1	2.028378612	1.23969E-06
KLF15	2.01322809	5.78655E-13
HIST2H2BE	2.007082142	3.72819E-21
KCNG1	1.99597935	2.07834E-35
GUCY1A2	1.981558617	2.09226E-32
CES4A	1.976631755	6.14889E-13
SH2D6	1.976258856	1.10393E-05
ADPRHL1	1.971400604	6.34602E-38
EYA4	1.965449016	4.66832E-62
ALDH3B1	1.957676219	2.46929E-68
PRR5L	1.953457948	6.06672E-90
FO681492.1	1.949850881	3.59379E-07
INHBE	1.948535512	1.92217E-06
GJA5	1.946559183	1.09809E-05

Supplemental Table S6: Differentially expressed proteins in OVCA5 cells co-cultured with adipocytes vs monoculture identified by RPPA.

Protein	FC	p-Value
MUC1	4.569907768	0.006117043
HLA-DQA1	2.151034024	0.002293898
KDR	1.673480304	0.001440737
POLG	1.660980919	0.035461283
MAPK11/12/13/14	1.606703671	0.046223318
PXN	1.370352797	0.00204821
EPHA2	1.331991427	0.036156461
MAP2K1	1.324561255	0.000940074
ACSL1	1.298096482	0.020764282
ACACA-B	1.259539088	0.037767084
ARHGAP45	1.239485261	0.033462747
AKT2	1.203454388	0.00114316
RPS6KB1	1.181132878	0.00172152
GZMB	1.178366705	0.006491546
GSK3A/GSK3B	1.174894689	0.03061993
MAP1LC3A/B	1.171015988	0.041545454
MSI2	1.166803439	0.027602672
SRC	1.163325966	0.040191298
GLS	1.16237204	0.027346867
EIF4E	1.153529515	0.003149807
MYH9	1.148826964	0.026751327
EIF4G1	1.148683471	0.037335561
RBM15	1.142732509	0.036345095
CASP7	1.140853417	0.034995757
PTPN11	1.140023599	0.019174232
ERBB2	1.139701357	0.02458184
UVRAG	1.137095366	0.027310444
YBX1	1.13553631	0.009917987
RPS6KB1	1.129462184	0.000799302
AKT1/2/3	1.128347421	0.022429877
CDK1-3	1.125899434	0.043471572
BABAM1	1.121321478	0.004693334
EGFR	1.120562092	0.009931572
MLKL	1.120173457	0.007740228
WWTR1	1.117828208	0.001244278
SERPINE1	1.099194356	0.046845593
FGF2	1.097236826	0.042072565
PMS2	1.090053596	0.007141664
ATG3	1.08441346	0.048549321

BIRC3	1.081597268	0.001481759
GRB2	1.072598603	0.001876908
MMP2	1.068569415	0.016300495
CHEK1	1.041899051	0.028390664
PTCH1	1.038626034	0.042606428
RIPK3	1.032046587	0.035296859
RAD51	0.980611293	0.042958354
H2AX	0.976391148	0.00781176
BECN1	0.974645845	0.009119022
ZAP70	0.970738581	0.017636358
E2F1	0.956065721	0.045162944
FANCD2	0.953778759	0.045933155
FOXO3	0.940564079	0.02691957
AMBRA1	0.940185499	0.012533196
HSPB1	0.936637697	0.029988764
GAB2	0.927532877	0.013461028
PAK4	0.921395708	0.041561935
RAB11FIP1	0.915754185	7.57943E-06
PRKAR1A	0.905363032	0.022563446
SOX7	0.901241868	0.014164625
MACC1	0.884730869	0.045507955
PKMYT1	0.884693208	0.034922183
KIT	0.852864417	0.03021418
EZH2	0.84458198	0.010601551
RPA2	0.837962947	0.002626832
SRC	0.815553989	0.008014572
GLI3	0.808387997	0.011583747
ERBB2	0.778071898	0.004183021
CANX	0.690172768	0.028249838
TRIM24	0.638097131	0.015793239
SGK1	0.534224592	0.006052946
PTGS2	0.514779889	0.001209832
DDR1	0.507870745	0.043306451
SIRPA	0.494742442	0.000529841
SNAI1	0.485712161	0.003890062
NR3C1	0.459422169	0.008861829
LCN2	0.439058466	0.002442653
STING1	0.251709971	0.000277548
