# Supplementary material for: Complement activation at the interface between adipocytes and cancer cells drives tumor progression
Source: JCI Insight. 2025 Feb 18;10(6):e184935. doi: 10.1172/jci.insight.184935 (PMC11949041; doi:10.1172/jci.insight.184935)

Figure 3F

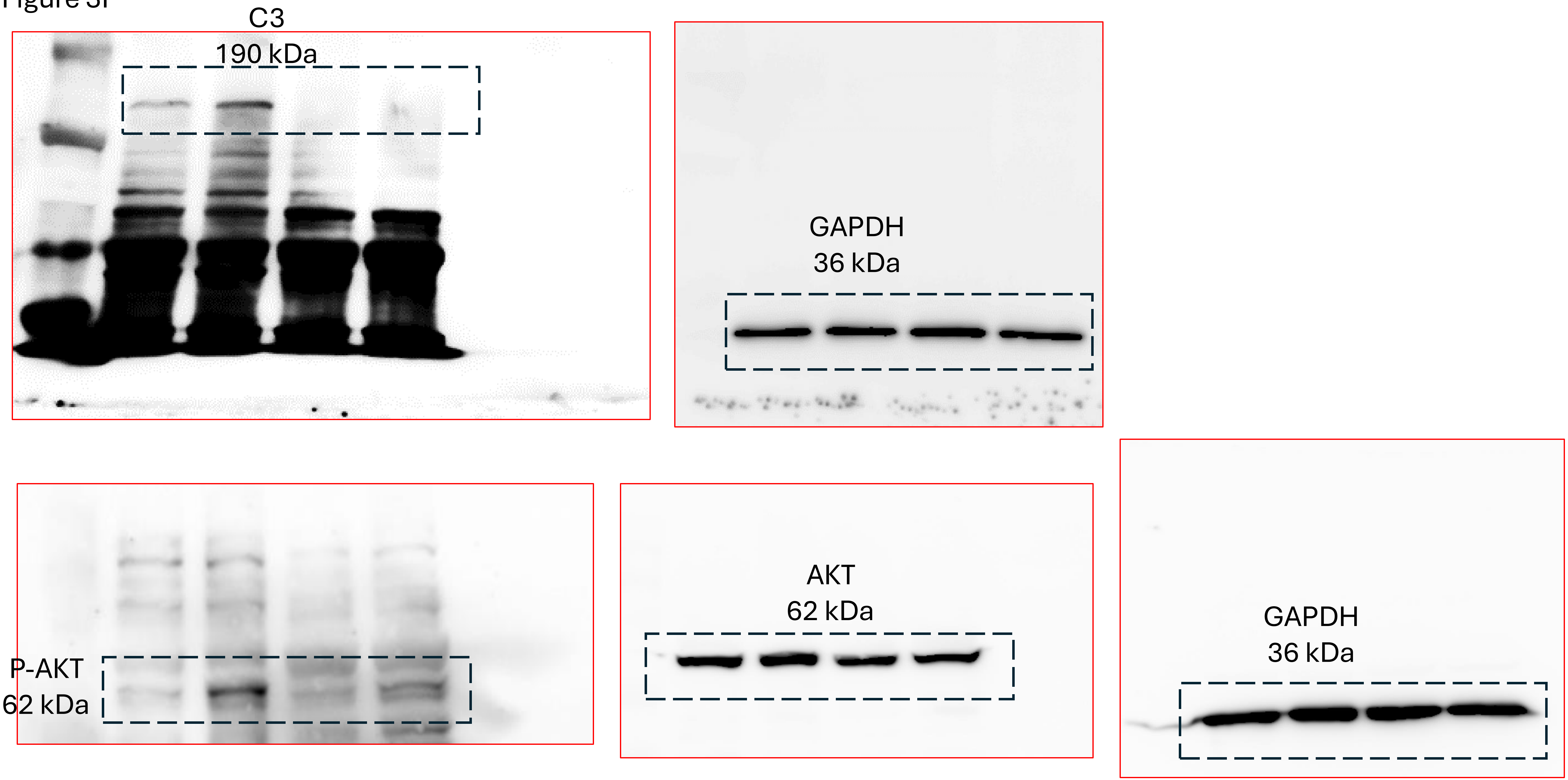

Figure 3J

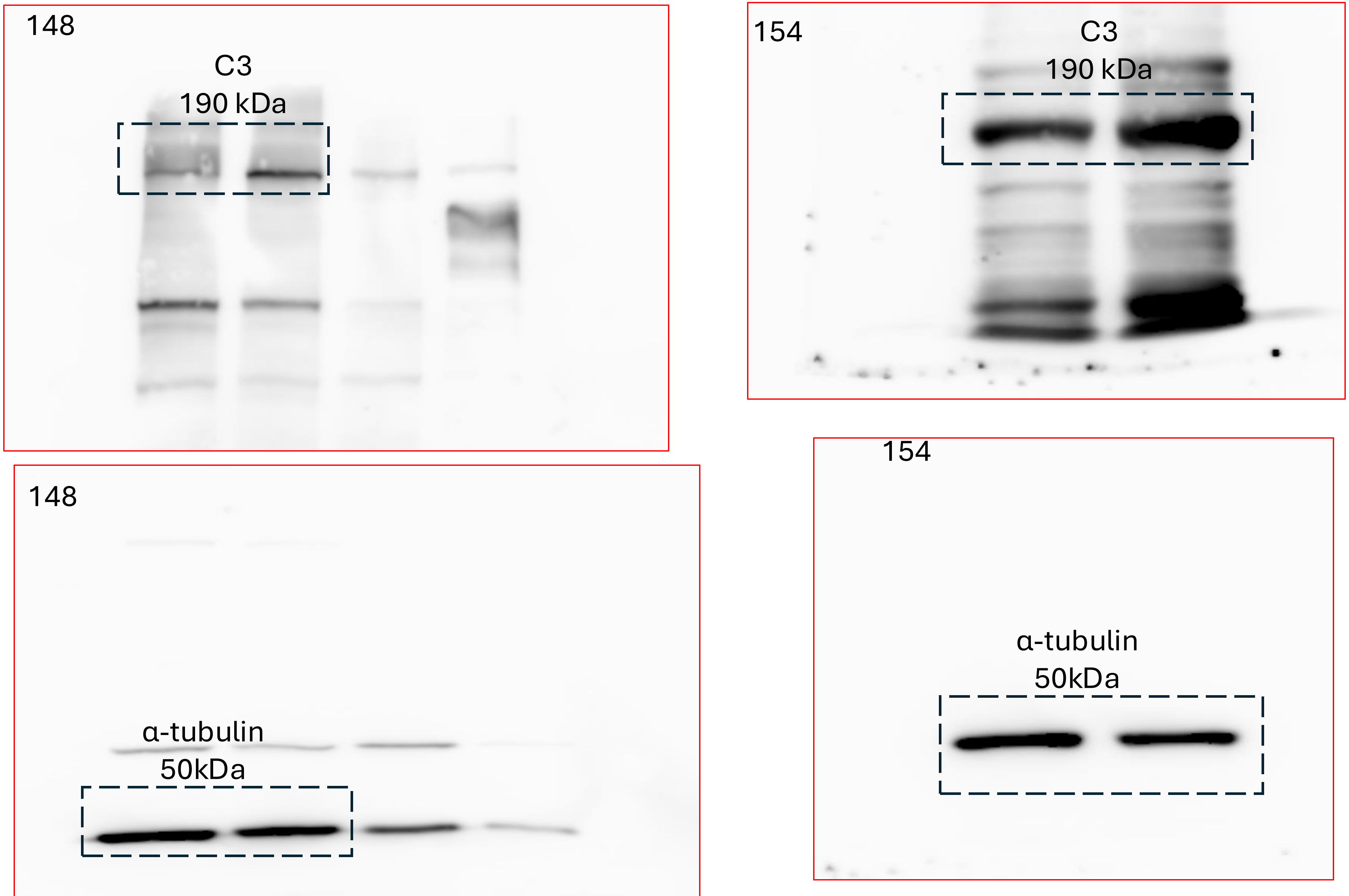
148
C3
190 kDa
154
C3
190 kDa
148
α-tubulin
50kDa
154
α-tubulin
50kDa

Figure 6G

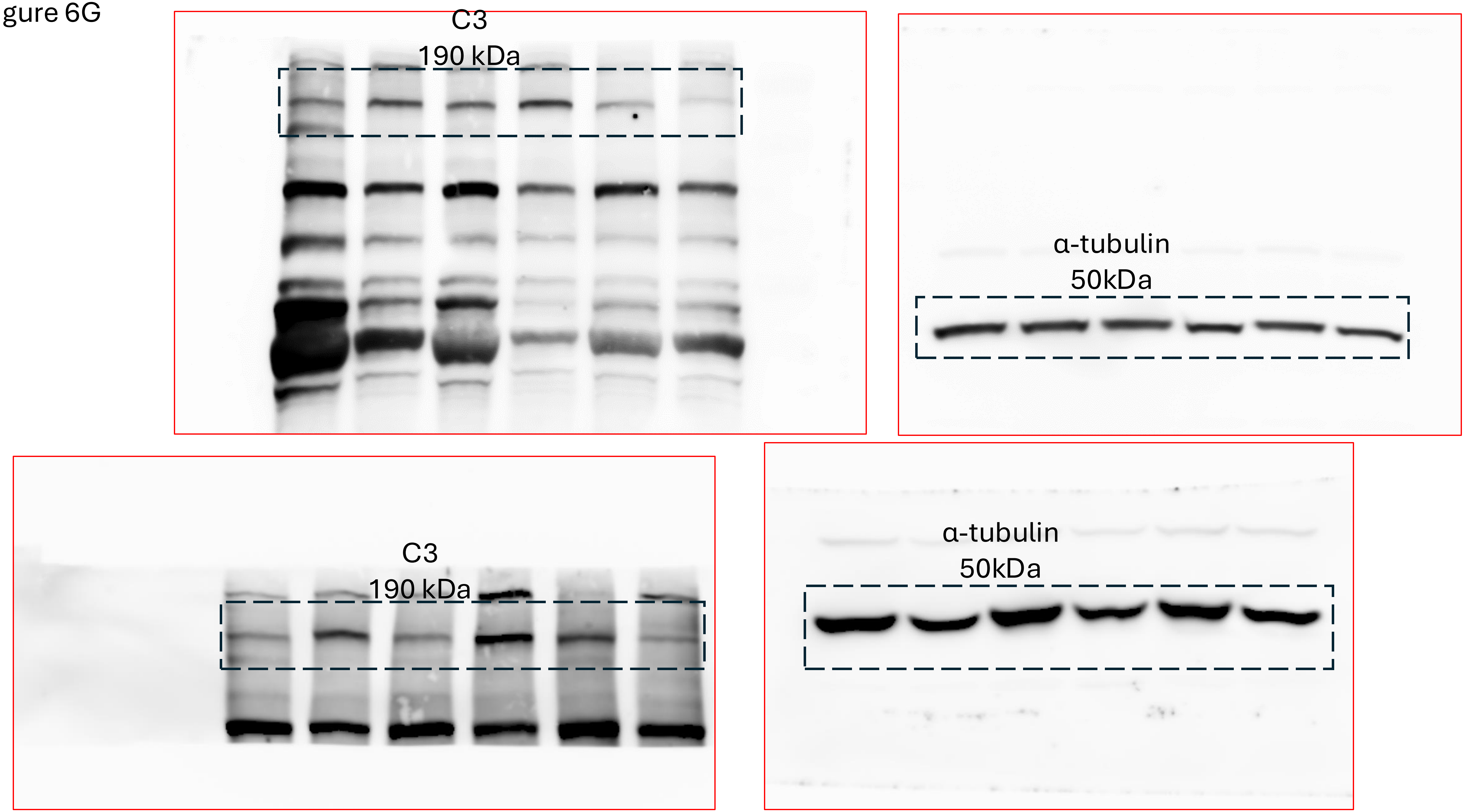

Figure 7D

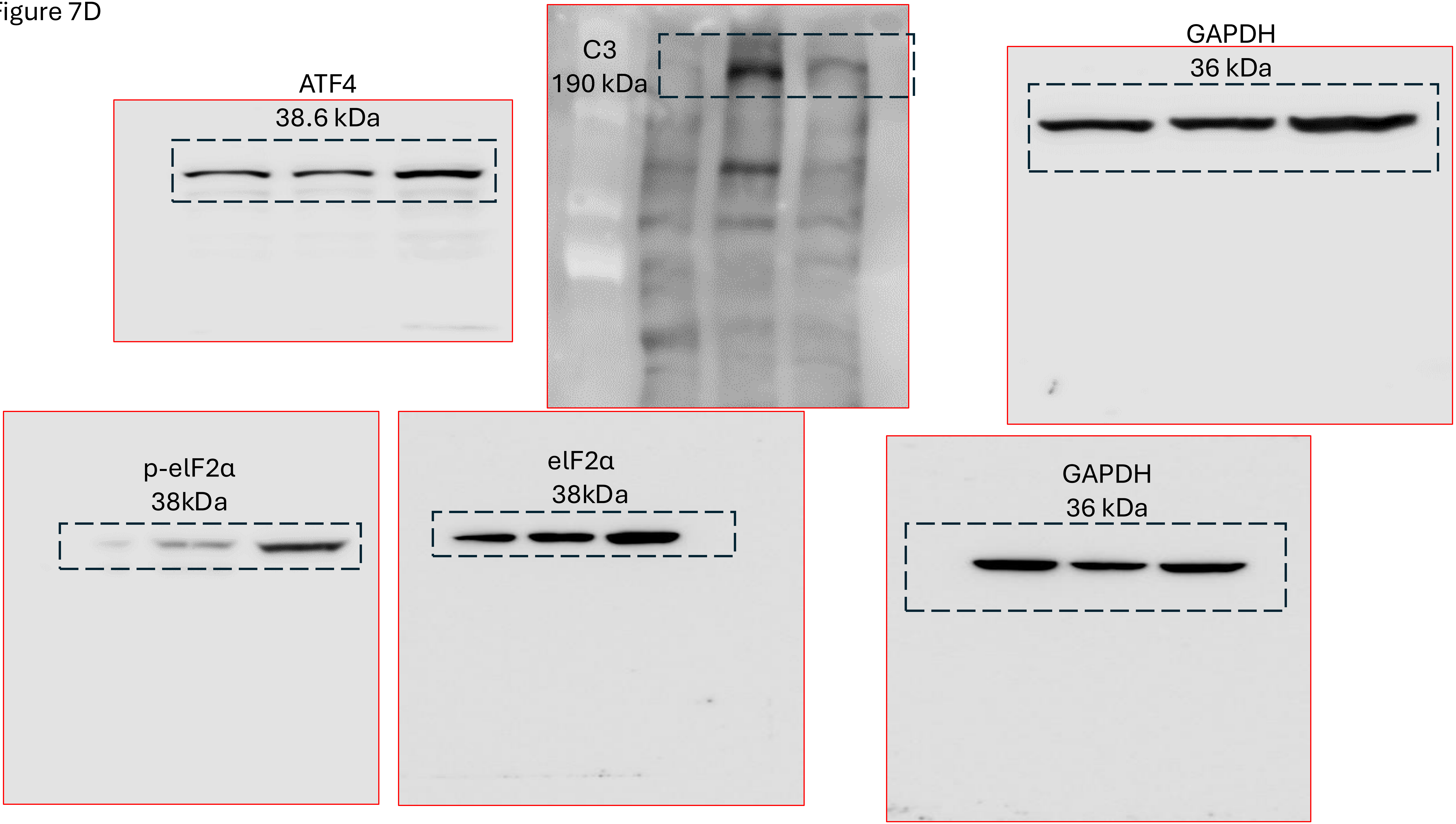
ATF4
38.6 kDa
C3
190 kDa
GAPDH
36 kDa
p-eIF2α
38kDa
eIF2α
38kDa
GAPDH
36 kDa

Figure 7E

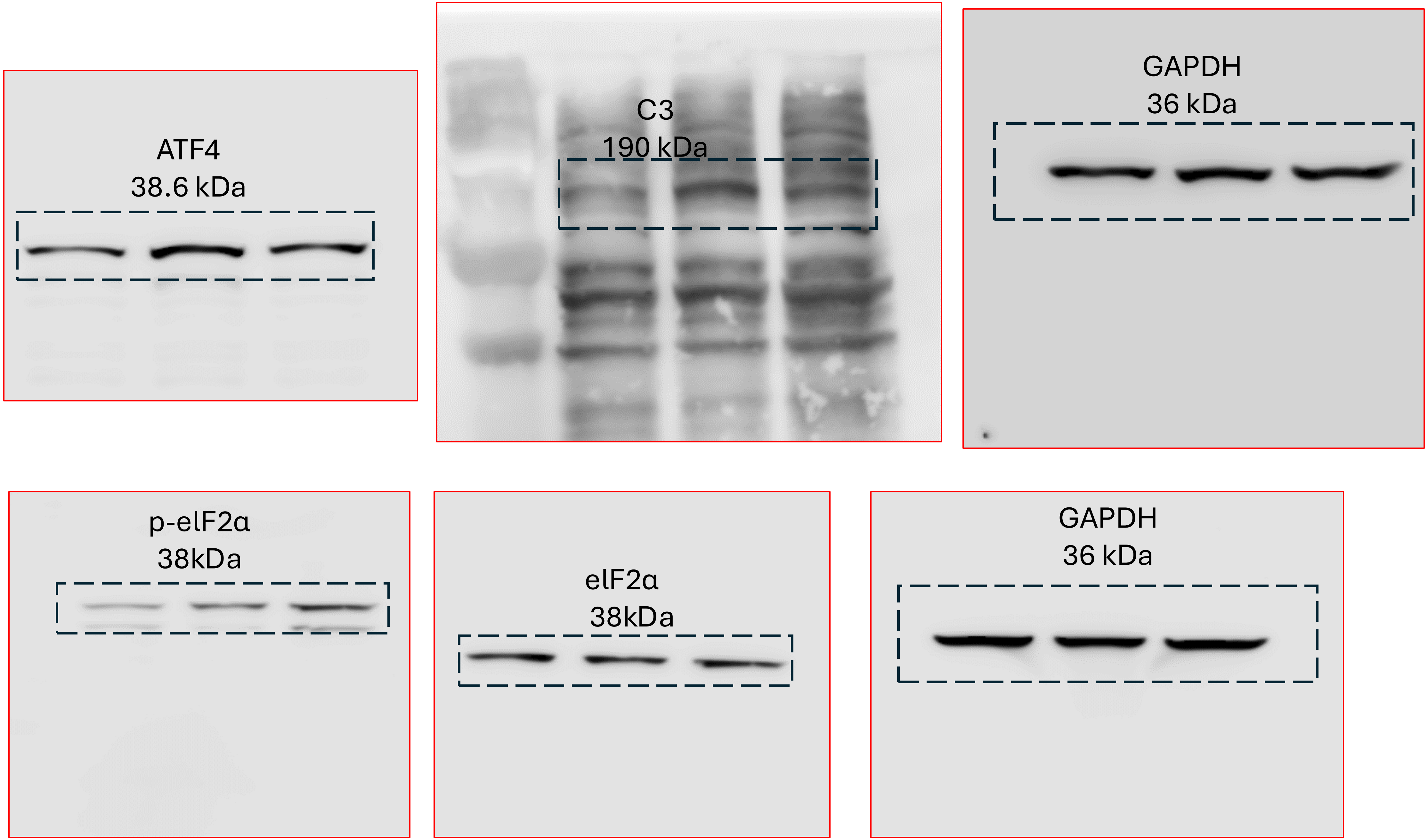
ATF4
38.6 kDa
C3
190 kDa
GAPDH
36 kDa
p-elF2α
38kDa
elF2α
38kDa
GAPDH
36 kDa

Figure 7G

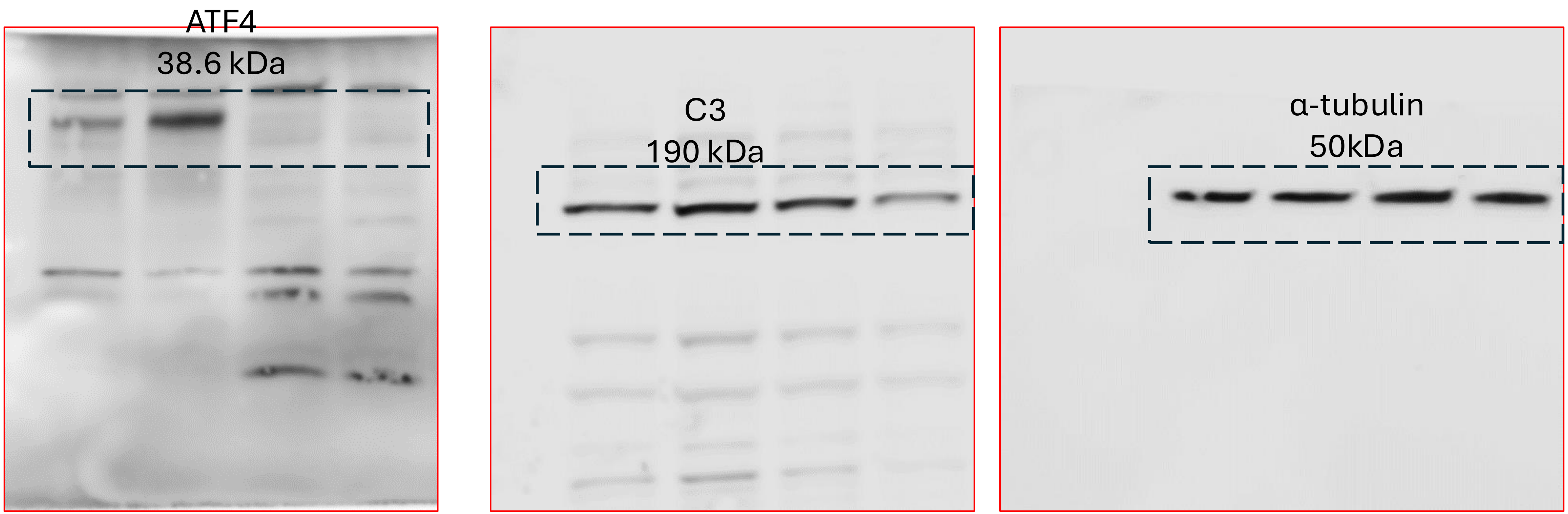

Supp Figure 2D

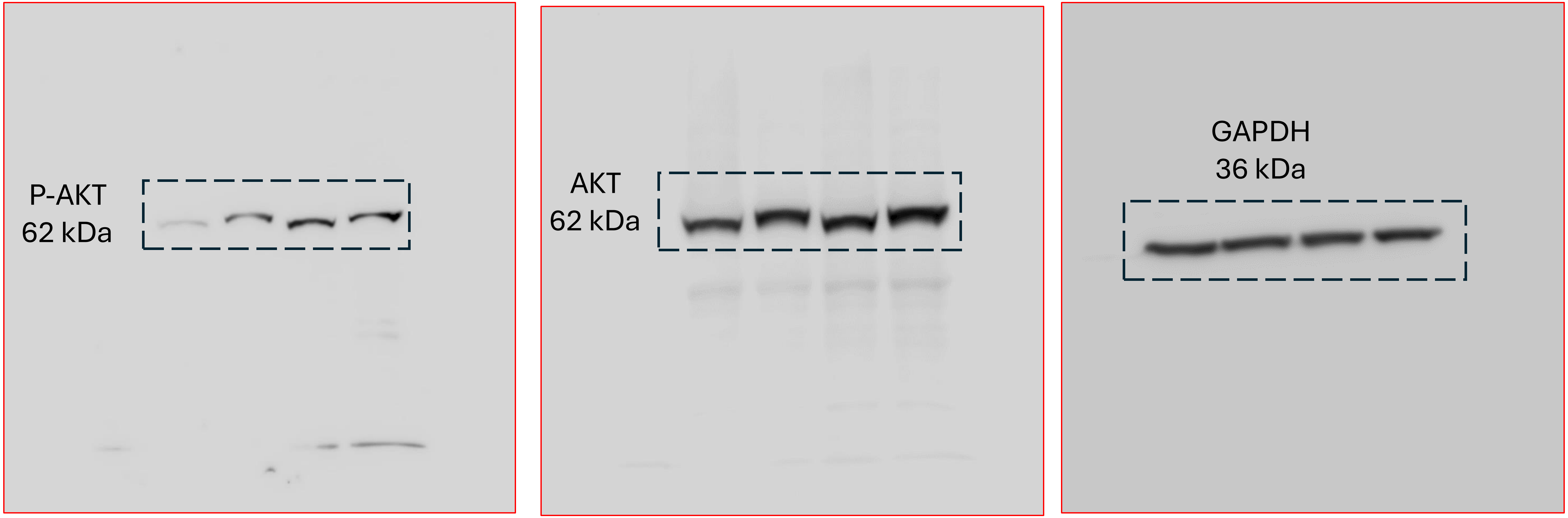

Supp Figure 4E

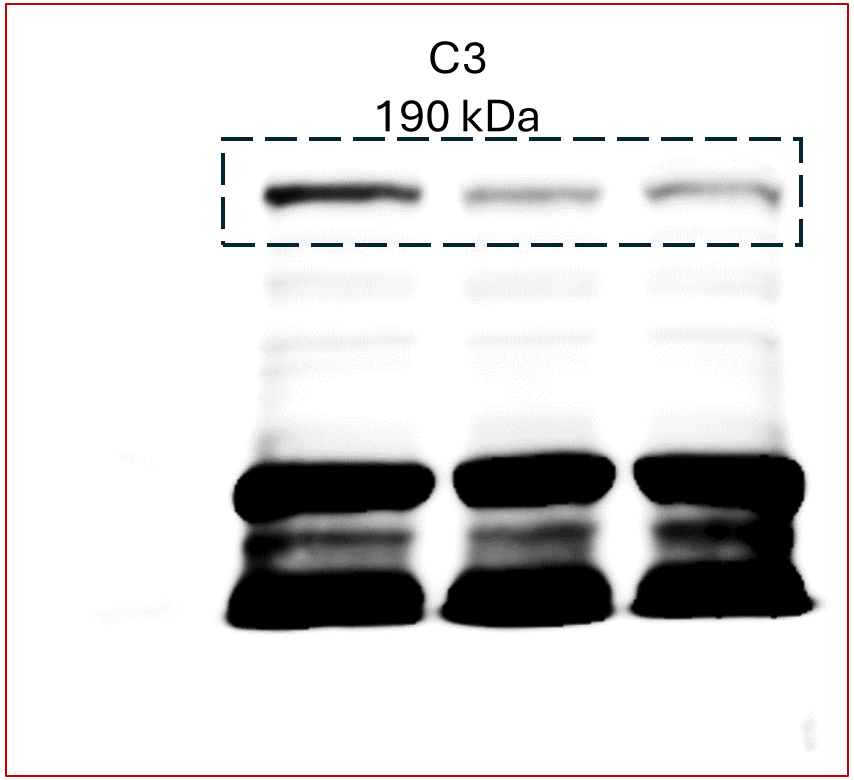

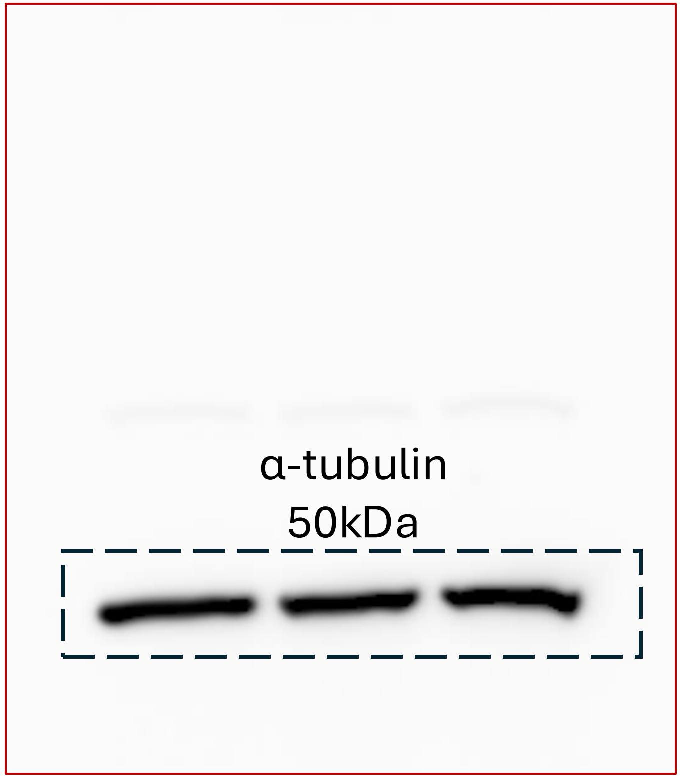

Supplement: Unedited blot and gel images [file jciinsight-10-184935-s131.pdf]
